# Sample size calculation for estimating key epidemiological parameters using serological data and mathematical modelling

**DOI:** 10.1186/s12874-019-0692-1

**Published:** 2019-03-07

**Authors:** Stéphanie Blaizot, Sereina A. Herzog, Steven Abrams, Heidi Theeten, Amber Litzroth, Niel Hens

**Affiliations:** 1 Centre for Health Economics Research and Modelling Infectious Diseases (CHERMID), Vaccine and Infectious Disease Institute (VAXINFECTIO), University of Antwerp, Antwerp, Belgium; 2 Institute for Medical Informatics, Statistics and Documentation, Medical University of Graz, Graz, Austria; 3 Interuniversity Institute for Biostatistics and statistical Bioinformatics, UHASSELT, Hasselt University, Hasselt, Belgium; 4 Centre for the Evaluation of Vaccination, Vaccine and Infectious Disease Institute (VAXINFECTIO), University of Antwerp, Antwerp, Belgium; 5 Service of Epidemiology of infectious diseases, Scientific Directorate Epidemiology and Public Health, Sciensano, Brussels, Belgium

**Keywords:** Infectious diseases, Mathematical models, Study design, Sample size, Allocation, Precision

## Abstract

**Background:**

Our work was motivated by the need to, given serum availability and/or financial resources, decide on which samples to test in a serum bank for different pathogens. Simulation-based sample size calculations were performed to determine the age-based sampling structures and optimal allocation of a given number of samples for testing across various age groups best suited to estimate key epidemiological parameters (e.g., seroprevalence or force of infection) with acceptable precision levels in a cross-sectional seroprevalence survey.

**Methods:**

Statistical and mathematical models and three age-based sampling structures (survey-based structure, population-based structure, uniform structure) were used. Our calculations are based on Belgian serological survey data collected in 2001–2003 where testing was done, amongst others, for the presence of Immunoglobulin G antibodies against measles, mumps, and rubella, for which a national mass immunisation programme was introduced in 1985 in Belgium, and against varicella-zoster virus and parvovirus B19 for which the endemic equilibrium assumption is tenable in Belgium.

**Results:**

The optimal age-based sampling structure to use in the sampling of a serological survey as well as the optimal allocation distribution varied depending on the epidemiological parameter of interest for a given infection and between infections.

**Conclusions:**

When estimating epidemiological parameters with acceptable levels of precision within the context of a single cross-sectional serological survey, attention should be given to the age-based sampling structure. Simulation-based sample size calculations in combination with mathematical modelling can be utilised for choosing the optimal allocation of a given number of samples over various age groups.

**Electronic supplementary material:**

The online version of this article (10.1186/s12874-019-0692-1) contains supplementary material, which is available to authorized users.

## Background

Several key epidemiological parameters such as the prevalence, the force of infection – rate at which susceptible individuals become infected, or the basic reproduction number R_0_ – expected number of secondary cases of an infected person in a totally susceptible population – can be computed through the use of mathematical models.

Mathematical models for infectious diseases often rely on data from serological surveys. Specifically, in a cross-sectional serological survey, samples taken from individuals at a certain time point provide information about whether or not these individuals have been immunised before that time point (depicting current status data). Pathogen-specific antibodies following infection or vaccination can be identified in the serum. The antibody levels are typically compared to a predetermined cut-off level to determine the individuals’ humoral immunological status. The usefulness of these surveys in epidemiology has recently been highlighted [1]. Under the assumptions of lifelong humoral immunity and an epidemic in a steady state, the age-specific force of infection can be estimated from such data [2].

Publications that reported using a dynamic transmission model to inform the design of studies in infectious diseases are scarce [3]. Moreover, only a few studies used mathematical or statistical models to inform the design of serological surveys. Marschner [4] introduced a method for determining the sample size of a cross-sectional seroprevalence survey to estimate the age-specific incidence of an irreversible disease, based on the illness-death model assuming time homogeneity and non-differential mortality as described in Keiding’s 1991 paper [5]. More recently, Nishiura et al. [6] proposed a framework to compute the uncertainty bounds of the final epidemic size to H1N1–2009 and to determine the minimum sample size required. Sepúlveda and Drakeley [7] proposed two sample size calculators, depending on whether the seroreversion rate (i.e., rate of antibody decay) is known, for estimating the seroconversion rate in malaria transmission in low endemicity settings using a reverse catalytic model. They extended the method to determine the sample size required to detect a reduction in the seroconversion rate at a given time point before survey sampling caused by a field intervention [8]. Lastly, Vinh and Boni [9] assessed the power of serial serological studies in inferring the basic reproduction number and other processes of influenza using a mathematical model.

In this paper, simulation-based sample size calculations are performed in order to determine the age-based sampling structures and optimal allocation distributions best suited to estimate with acceptable precision levels several epidemiological parameters such as the prevalence, force of infection, and basic reproduction number. Specifically, we use four models and three age-based sampling structures within the context of a single cross-sectional seroprevalence survey. We differentiate between endemic and non-endemic settings. In the latter case, we limit ourselves to estimating the prevalence and defer extensions thereof to future work. The objectives of this paper are i) to give insights into the age structure best suited to estimate the parameters with acceptable levels of precision; ii) to provide an order of magnitude of the sample size required to attain a specified precision for a particular parameter; and iii) to give insights into the optimal allocation of a fixed sample size among age groups.

Our work is motivated by the need to, given serum availability and/or financial resources, decide on which samples to test in a serum bank for different pathogens. In particular, the proportion of the samples to allocate in different age groups could be investigated to obtain the highest precision for a given parameter.

## Methods

### Data

A serological survey testing for the presence of, amongst others, measles, mumps, rubella, varicella-zoster virus (VZV), and parvovirus B19 Immunoglobulin G (IgG) antibodies was conducted on large representative national serum banks in Belgium [10]. Serum samples were collected, between 2001 and 2003, from residual blood samples used for routine laboratory testing (individuals aged < 18 years) or from blood donors (18 years and over). This survey was designed as proposed by the European Sero-Epidemiology Network (ESEN) which aimed to standardize the serological surveillance of immunity to various diseases in European countries [11]. In particular, children and adolescents were oversampled in the survey. A total of 3378 samples were collected and the age of the individuals ranged from 0 to 65 years. The number of samples with immunological status with regard to measles, mumps, rubella, VZV, and parvovirus B19 infections were 3190, 3004, 3195, 3256, and 3080, respectively. Since a national immunisation programme against measles, mumps, and rubella has been introduced in 1985 in Belgium with gradually increasing vaccine coverage in the targeted age groups (infants, adolescents aged 11–13 years, and catch-up campaigns in adults), endemic equilibrium for these infections in 2002 cannot be assumed. In contrast, no immunisation programme against VZV and parvovirus B19 has been introduced, making endemic equilibrium a tenable assumption for both infections.

### Models

Here, we briefly present an overview of the methods used to derive key epidemiological parameters from serological survey data and we refer to Hens et al. [2] for a more in-depth explanation of the methodology. We start from the basic concept of an age-specific prevalence and gradually move to the force of infection and other parameters such as the basic and effective reproduction numbers in endemic equilibrium.

Age-specific seroprevalence can be modelled in the framework of generalized linear models (GLMs). For example, the probability to be infected at (before) a given age can be modelled through a logistic model, expressing the dependency on age using a specific functional form (see e.g. Hens et al. [12]). For estimating the (age-specific) force of infection from seroprevalence data, various statistical methods have been used in the literature including linear and non-linear parametric (e.g., fractional polynomials or catalytic model) and non-parametric approaches. Complementarily, the flow of individuals between the mutually exclusive stages of an infectious disease can be described using compartmental dynamic transmission models. The simplest such model, the Susceptible-Infectious-Recovered (SIR) model, describes the flow between the susceptible (*S*), the infected and infectious (*I*), and the recovered class (*R*). The following set of partial differential equations in continuous age and time can be used to describe the SIR dynamics mathematically:

$$ \left\{\begin{array}{c}\frac{\partial S\left(a,t\right)}{\partial a}+\frac{\partial S\left(a,t\right)}{\partial t}=-\lambda \left(a,\mathrm{t}\right)S\left(a,t\right)-\mu \left(a,t\right)S\left(a,t\right),\\ {}\frac{\partial I\left(a,t\right)}{\partial a}+\frac{\partial I\left(a,t\right)}{\partial t}=\lambda \left(a,t\right)S\left(a,t\right)-\sigma \left(a,t\right)I\left(a,t\right)-\mu \left(a,t\right)I\left(a,t\right),\\ {}\frac{\partial R\left(a,t\right)}{\partial a}+\frac{\partial R\left(a,t\right)}{\partial t}=\sigma \left(a,t\right)I\left(a,t\right)-\mu \left(a,t\right)R\left(a,t\right),\end{array}\right. $$

with the age- and time-specific population size given by *N*(*a*, *t*) = *S*(*a*, *t*) + *I*(*a*, *t*) + *R*(*a*, *t*) and with *λ*(*a*, *t*) the force of infection, *σ*(*a*, *t*) the recovery rate, and *μ*(*a*, *t*) the all-cause mortality rate.

Assuming a closed population of size *N* in demographic and endemic equilibrium, we obtain a set of ordinary differential equations (ODEs):

$$ \left\{\begin{array}{c}\frac{dS(a)}{da}=-\lambda (a)S(a)-\mu (a)S(a),\\ {}\frac{dI(a)}{da}=\lambda (a)S(a)-\sigma (a)I(a)-\mu (a)I(a),\\ {}\frac{dR(a)}{da}=\sigma (a)I(a)-\mu (a)R(a).\end{array}\right. $$

Solving the above set of ODEs, the following expression for the seroprevalence of individuals of age *a* is obtained:

$$ \pi (a)=1-\mathit{\exp}\left(-{\int}_0^a\lambda (u) du\right). $$

The above equation can be solved numerically by using a discrete age class framework, thereby assuming a constant force of infection *λ*_*k*_ in each age class [*a*_[*k*]_, *a*_[*k* + 1]_], *k* = 1, ⋯, *J* [13]. The seroprevalence at age *a* in the *j*^th^ age interval is approximated by:

1 $$ \pi (a)=1-\mathit{\exp}\left(-\sum \limits_{k=1}^{j-1}{\lambda}_k\left({a}_{\left[k+1\right]}-{a}_{\left[k\right]}\right)-{\lambda}_j\left(a-{a}_{\left[j\right]}\right)\right), $$

where *a*_[1]_ = 0 and *a*_[*J* + 1]_ = *L* (the life expectancy).

This model assumes that the infection-related mortality can be neglected, which is tenable for the infections studied in the present paper, and that the total population size is constant over time (i.e. the number of births and deaths are balanced) with a constant age distribution.

From this model, other key epidemiological parameters can be calculated such as the basic and effective reproduction number (R_0_ and R_eff_ respectively; R_eff_ reflects the actual average number of secondary cases that can be observed in a partially immune population) or the average age at infection.

Since seropositive results for measles, mumps, and rubella are a mix of vaccine- and infection-induced immunity, implying time-heterogeneity which is beyond the scope of this paper, only the age-specific seroprevalence for these diseases was modelled. We considered a logistic model with piecewise constant prevalence values within the following age classes based (partially) on vaccination policies: [1,2), [2,11), [11,16), [16,21), [21,31), and [31,65] years. The estimates of the coefficients using this model (on the logit scale) are denoted by $\widehat{\beta}$.

For VZV and parvovirus B19 infections, for which an endemic equilibrium is tenable in Belgium, three mathematical models for estimating the force of infection, used in previous studies [2, 14–16], were considered.

The first model is a Maternally-derived immunity-Susceptible-Infectious-Recovered (MSIR) model with piecewise constant force of infection. An MSIR compartmental model adds to the basic SIR model a stage describing newborns and infants protected by maternally acquired immunity (class *M*) [17]. This model assumes that newborns and infants are protected by maternal antibodies and that this immunity is promptly lost at a given age *A*. Newborns and infants are then assumed to be susceptible to infection (class *S*), they may become infected and infectious (class *I*), before recovering from the infection (class *R*). The seroprevalence at age *a* in the *j*^th^ age interval is approximated by, which is a slight adaptation of the model in (Eq. 1):

$$ \pi (a)=1-\mathit{\exp}\left(-\sum \limits_{k=1}^{j-1}{\lambda}_k\left({a}_{\left[k+1\right]}-{a}_{\left[k\right]}\right)-{\lambda}_j\left(a-{a}_{\left[j\right]}\right)\right), $$

with *a*_[1]_ = *A*, where *A* is the age at which maternal immunity is lost. In this paper, we considered an MSIR model with piecewise constant force of infection within the following six age classes: [1,2), [2,6), [6,12), [12,19), [19,31), and [31,65] years. These age groups account for the Belgian schooling system, the fact that infection mainly takes place among young age groups, and for which similar forces of infection are expected between ages in a given age group. Note that, in general, the choice of these age categories is often done on an ad-hoc basis.

The second model considered in this paper is the exponentially damped model for the force of infection as described by Farrington [14]. This model is based on the typical shape of the age-specific force of infection of childhood infectious diseases: low at birth because of the presence of maternal antibodies, then increasing linearly with age, and finally declining from a certain age onwards by an exponential decrease. The force of infection can be formulated as follows:

$$ \lambda (a)=\left({\alpha}_1a-{\alpha}_3\right){e}^{-{\alpha}_2a}+{\alpha}_3, $$

with *α*_1_, *α*_2_ and *α*_3_ the model parameters to be estimated from the data. Integrating *λ*(*a*) results in a non-linear model for the seroprevalence, i.e.,

$$ \pi \left(\mathrm{a}\right)=1-\mathit{\exp}\left\{\frac{\alpha_1}{\alpha_2}a{e}^{-{\alpha}_2a}+\frac{1}{\alpha_2}\left[\frac{\alpha_1}{\alpha_2}-{\alpha}_3\right]\left[{e}^{-{\alpha}_2a}-1\right]-{\alpha}_3a\right\}. $$

We considered a third model for parvovirus B19 infection, a mathematical model allowing for boosting and waning immunity, since lifelong protection against infection upon recovery from parvovirus B19 is questionable [18–22], which would limit the use of the two previous models. Goeyvaerts et al. [16] investigated several extensions of the MSIR model to determine whether waning of disease-acquired antibodies and/or boosting of low immunity by exposure to infectious individuals should be accounted for. Here, we used the model with the best Akaike information criterion (AIC) value which was the compartmental model allowing for age-specific waning of disease-acquired antibodies and boosting of low immunity, denoted by “MSIRWb-ext AW” (see Additional file 1). More specifically, waning was modelled using an additional state (*W*) with age-specific rates: individuals moved from the high immunity state *R* to the low immunity state *W* at a rate *ε*_1_ and *ε*_2_ for age group < 35 and ≥ 35 years respectively. The boosting rate was assumed to be proportional to the force of infection by a factor of φ. The transmission rates were assumed to be directly proportional to age-specific rates of making social contact with a proportionality factor *q*.

Samples from children aged less than 1 year (6 months in the MSIRWb-ext AW model to be consistent with the original article) were omitted in our analyses because of distortions expected from the presence of maternal antibodies against the various pathogens and low number of samples of that age (*n* = 13).

The first two columns of Table 1 show a summary of the models used for each of the pathogens studied. Formulas to calculate the various epidemiological parameters (i.e., age-standardized seroprevalence and force of infection, R_0_, R_eff_, and the average age of infection) can be found in Additional file 1. The age-specific seroprevalence and force of infection were calculated in the following age groups: [1,2), [2,6), [6,12), [12,19), [19,31), and [31,65] years for each pathogen (including measles, mumps, and rubella for easier reading).

**Table 1:** Summary of the models considered for each of the pathogens and the corresponding model parameter estimates using the observed serological survey data

Serological data	Models	Estimates
Measles	Logistic model with piecewise constant prevalence	${\widehat{\beta}}_{Measles}=\left(0.108,1.733,1.412,1.819,2.479,3.863\right)$
Mumps	Logistic model with piecewise constant prevalence	${\widehat{\beta}}_{Mumps}=\left(-0.575,1.317,1.990,1.950,2.145,2.112\right)$
Rubella	Logistic model with piecewise constant prevalence	${\widehat{\beta}}_{Rub}=\left(0.050,1.912,2.356,2.419,3.099,3.339\right)$
VZV	MSIR piecewise constant force of infection	${\widehat{\lambda}}_{VZV}=\left(\mathrm{0.330,0.301,0.245,0},\mathrm{0.071,0.116}\right)$
	Exponentially damped model for force of infection	${\widehat{\alpha}}_{VZV}=\left(\mathrm{0.476,0.468,0.071}\right)$
Parvovirus B19	MSIR piecewise constant force of infection	${\widehat{\lambda}}_{B19}=\left(\mathrm{0.065,0.086,0.114,0.036,0},0.014\right)$
	Exponentially damped model for force of infection	${\widehat{\alpha}}_{B19}=\left(\mathrm{0.076,0.241,0.006}\right)$
	MSIR model with boosting and waning (MSIRWb-ext AW)	$\widehat{q}=0.085$, ${\widehat{\varepsilon}}_1=0.012$, ${\widehat{\varepsilon}}_2=0$, and $\widehat{\varphi}=0.334$.

*VZV* varicella-zoster virus, *MSIR model* Maternally-derived immunity-Susceptible-Infectious-Recovered model. $\widehat{\beta}$: coefficient estimates (logit scale) within the age classes [1,2), [2,11), [11,16), [16,21), [21,31), and [31,65] years. $\widehat{\lambda}$: estimates of the force of infection within the age classes [1,2), [2,6), [6,12), [12,19), [19,31), and [31,65] years. $\widehat{\alpha}$: estimates of the three parameters describing the exponentially damped model. $\widehat{q}$: estimated proportionality factor between the transmission and contact rates; $\widehat{\varepsilon_1}$ and $\widehat{\varepsilon_2}$: estimated rates at which individuals moved from the high immunity state *R* to the low immunity state *W* for age group < 35 and ≥ 35 years respectively; *φ*: estimated proportionality factor between the boosting rate and the force of infection. See the Models section for more details

### Estimating the model parameters

Maximum likelihood estimates were obtained for each model and pathogen assuming that the observed prevalence follows a binomial distribution. Using the estimated values of the parameters for each model and pathogen (with age values rounded down to integer values), age-specific “true” prevalence values were calculated which were used in the simulations (see next section).

### Simulations

Three age structures were compared: the age structure derived from the pathogen-specific data of the serological survey in which children and adolescents were oversampled (survey-based), the age structure of the Belgian population in 2003 (population-based) [23], and a uniform age structure (see Additional file 1: Figure S1 and Table S1).

**Fig. 1:**
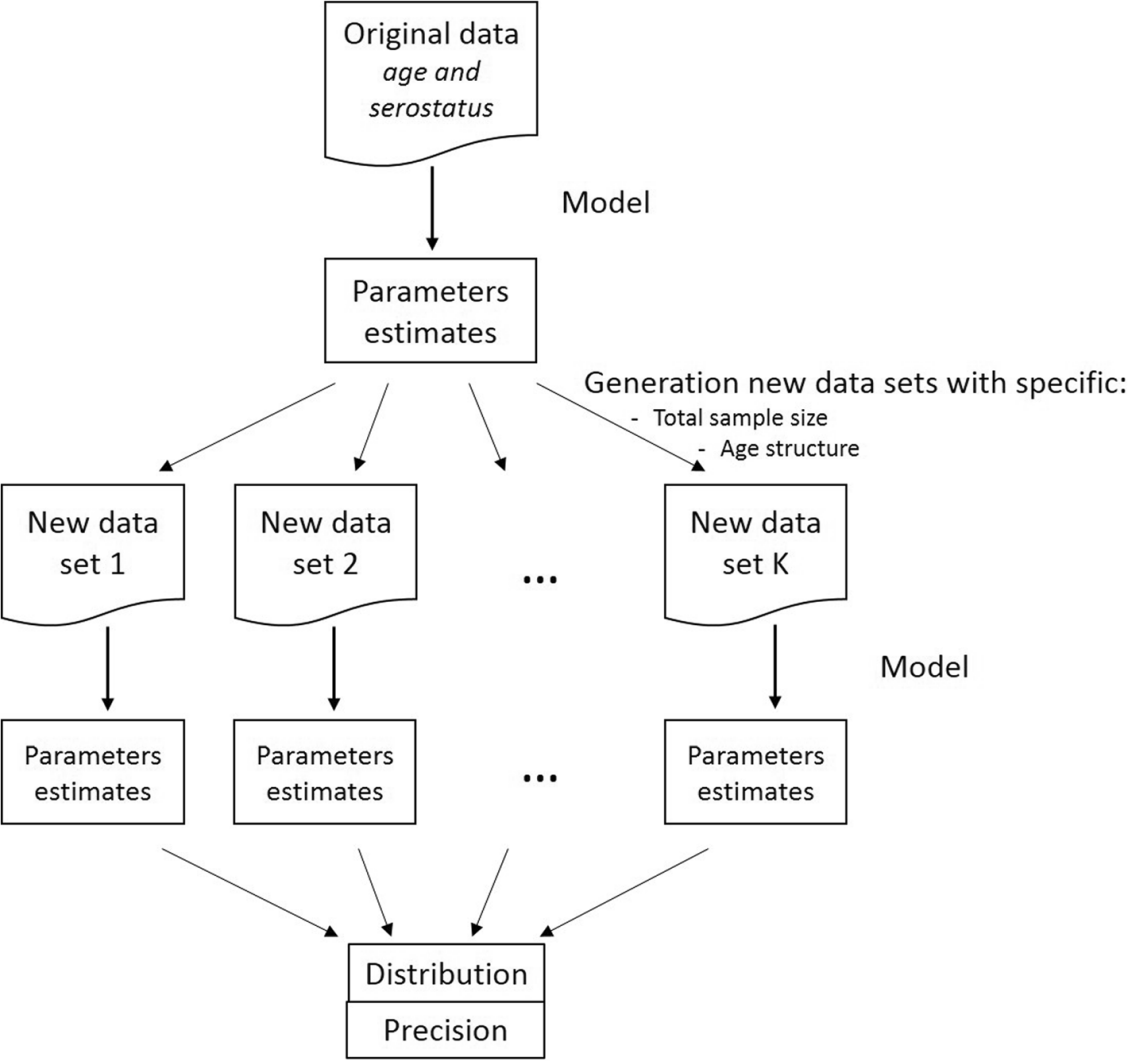
Schematic representation of the approach used in this paper

To compare the age-based sampling structures and determine the optimal allocation of samples over age groups, 500 (new) datasets were generated using a binomial distribution and the age-specific “true” prevalence values obtained for each model. We used several values of the total sample size (*N* = 1650, 3300, 6600, 9900, 13,200, or 19,800) and the number of samples across age depended on the age-based sampling structure or allocation distribution used. Each dataset was then fitted with the corresponding model to obtain a distribution of the parameters values and the precision. Here, the optimal allocation was determined by calculating the precisions obtained using different distributions. To restrict the number of distributions to compare, we varied the proportions among the six age groups ([1,2), [2,6), [6,12), [12,19), [19,31), and [31,65] years) from 10 to 50% (leading to 126 distributions) and assuming a uniform distribution within each age group. Figure 1 gives a schematic representation of the approach used in this paper. The precision was defined to be half the length of the 95% percentile-based confidence interval (CI) calculated over the 500 simulations. For the seroprevalence and force of infection by age group, the age distribution providing the best joint precision, defined as the sum of the precisions in each age group, is reported.

In the MSIR model with piecewise constant force of infection for the VZV infection, simulations with biologically implausible estimated values (> 10) were excluded; such values were obtained in the age group > 30 years due to a simulated prevalence of 100% in this age group. These simulations were replaced.

All analyses were performed using R software (version 3.3.1) [24].

## Results

### Estimates of the model parameters obtained using the observed serological survey data

The model estimates for each of the different pathogens are given in Table 1. Additional file 1: Figure S3 shows the estimated prevalence and force of infection for each model and disease. The models provided overall good fits and the results were close between the models. However, for parvovirus B19, the exponentially damped model was not able to capture the decrease in seroprevalence around age 30. In contrast, as expected, the MSIRWb-ext AW model was able to capture this decrease, albeit only partly. In this model, the force of infection had a bimodal shape (with modes around ages 7 and 35 years; Additional file 1: Figure S3).

Since our simulations were based on integer age values, the MSIR and MSIRWb-ext AW models were re-run after rounding continuous age values down to integers; however, the estimates were close when using continuous or integer values. The estimates obtained using the MSIR model with piecewise constant force of infection within the age classes [1,2), [2,6), [6,12), [12,19), [19,31), and [31,65] years were: ${\widehat{\lambda}}_{B19}=\left(\mathrm{0.077,0.104,0.100,0.035,0},0.014\right)$, ${\widehat{\lambda}}_{VZV}=\left(\mathrm{0.404,0.337,0.200,0},\mathrm{0.076,0.113}\right)$. The following estimates were obtained using the MSIRWb-ext AW model for parvovirus B19: $\widehat{q}=0.089$, $\widehat{\varepsilon_1}=0.014$, $\widehat{\varepsilon_2}=0$, and $\widehat{\varphi}=0.359$. Estimates of the prevalence and force of infection (overall and by age groups), R_0_, average age at infection, and R_eff_ are provided in Additional file 1: Tables S2-S4.

### Comparisons of the three age-based sampling structures

**Fig. 2:**
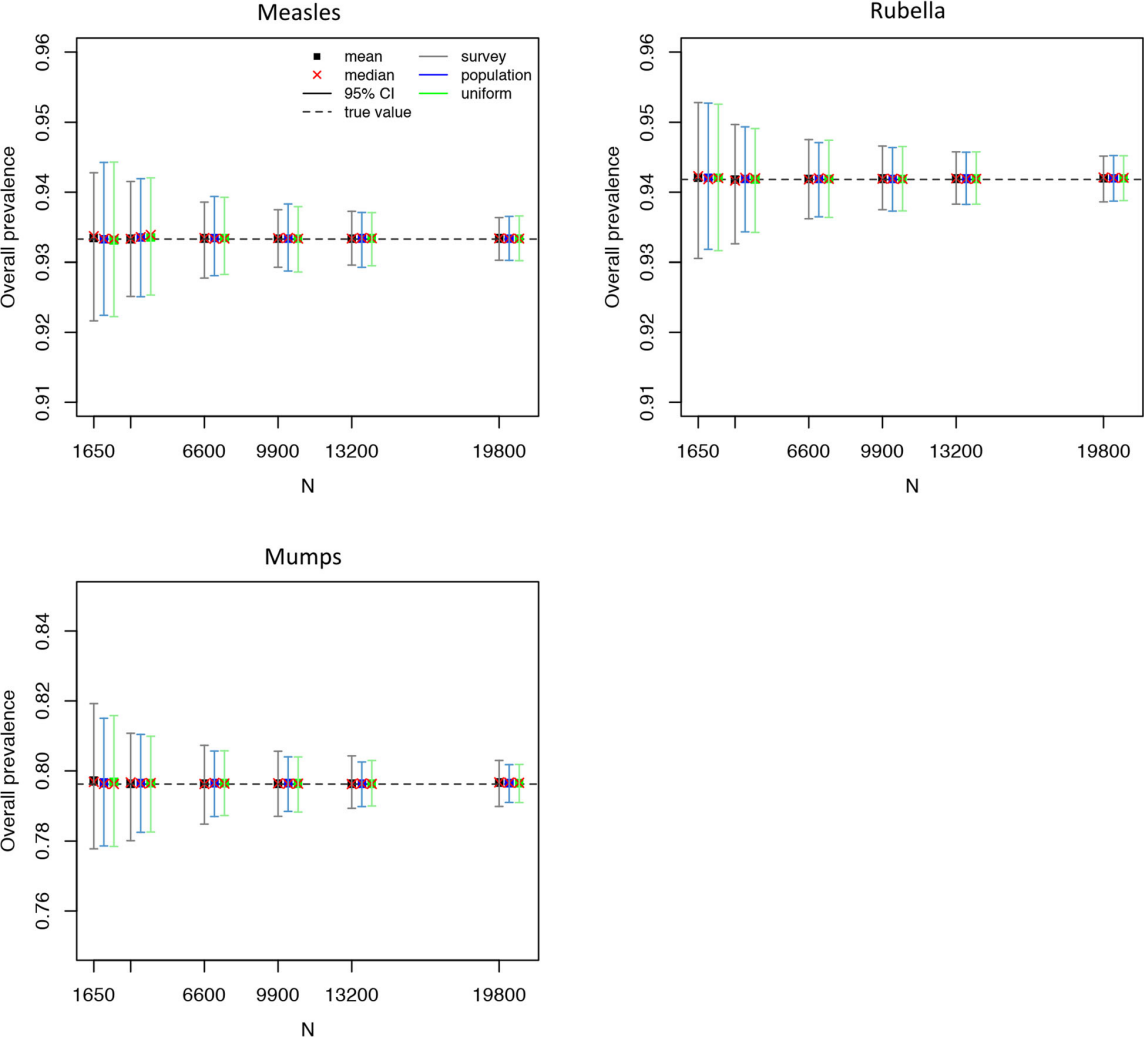
Measles, mumps, rubella serological data: mean, median, and 95% confidence interval for the overall seroprevalence over 500 simulations as a function of the total number of sampled individuals (N) using the logistic model with piecewise constant prevalence. Top left: Measles. Top right: Rubella. Bottom: Mumps. “True” overall seroprevalence is the estimated overall seroprevalence using the models on the observed serological survey data (with integer age values). The y-axes have different ranges of values for better legibility

**Fig. 3:**
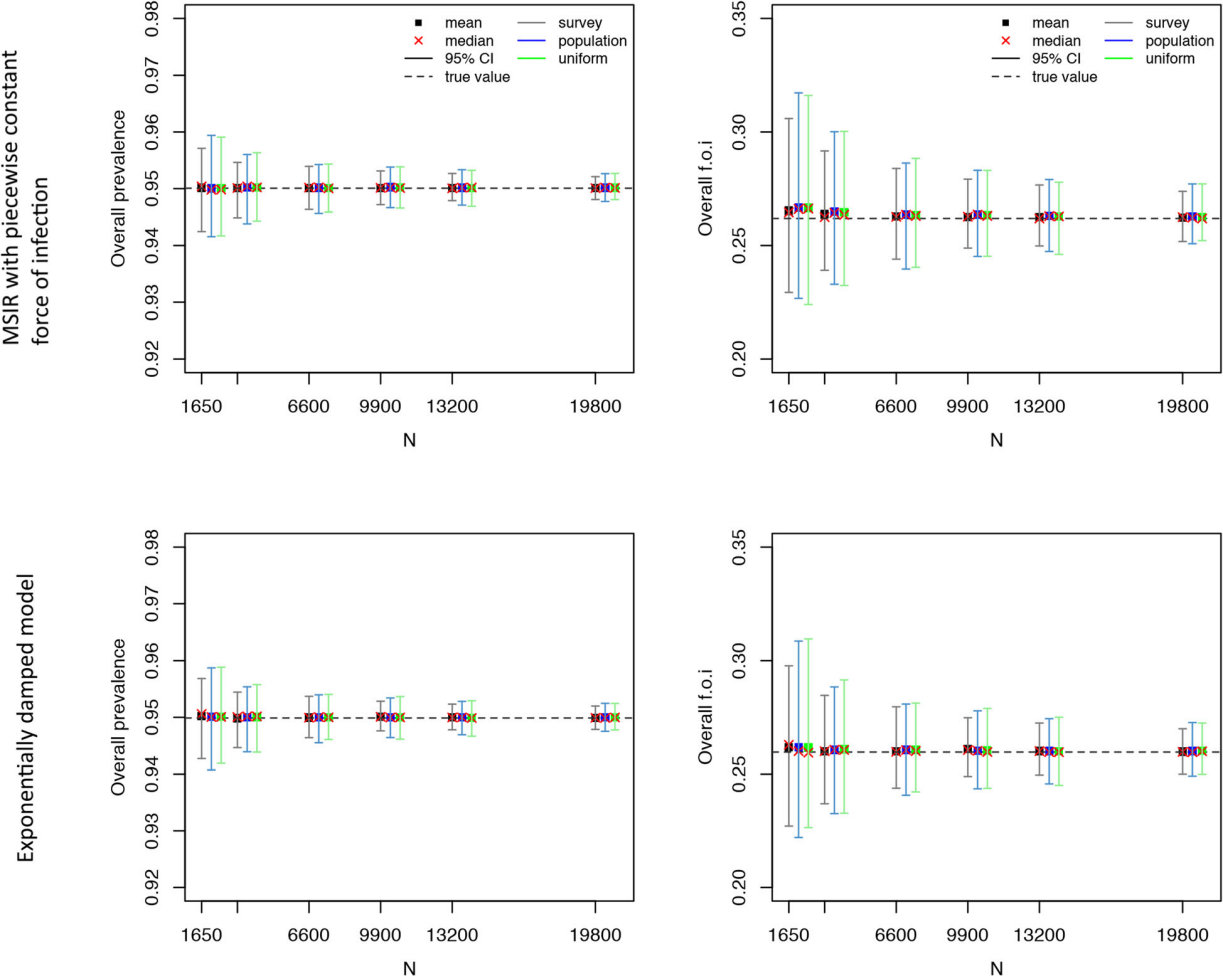
Varicella-zoster virus serological data: mean, median, and 95% confidence interval for the overall seroprevalence (left) and overall force of infection (right) over 500 simulations as a function of the total number of sampled individuals (*N*) for the Maternally-derived immunity-Susceptible-Infectious-Recovered (MSIR) model with piecewise constant force of infection (top) and the exponentially damped model (bottom). “True” overall seroprevalence is the estimated overall seroprevalence using the models on the observed serological survey data (with integer age values)

**Fig. 4:**
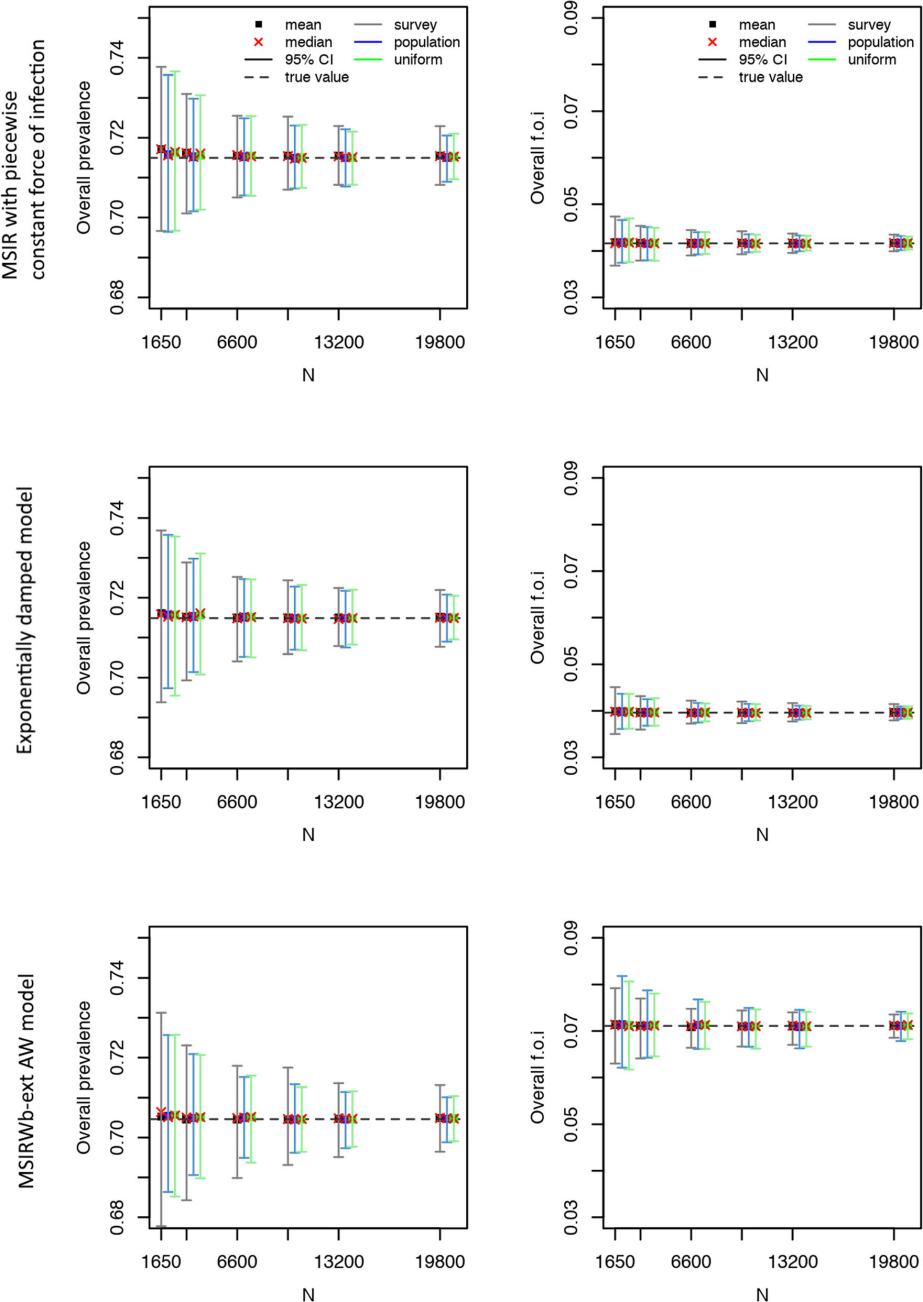
Parvovirus B19 serological data: mean, median, and 95% confidence interval for the overall seroprevalence (left) and overall force of infection (right) over 500 simulations as a function of the total number of sampled individuals (*N*) for the Maternally-derived immunity-Susceptible-Infectious-Recovered (MSIR) model with piecewise constant force of infection (top), the exponentially damped model (middle), and the MSIR model allowing for age-specific waning of disease-acquired antibodies and boosting of low immunity (MSIRWb-ext AW) model (bottom). “True” overall seroprevalence is the estimated overall seroprevalence using the models on the observed serological data (with integer age values)

Because similar results were obtained when generating 1000 or 1500 datasets, only the results based on 500 simulations are presented. For the overall seroprevalence of measles and VZV, in both models used, the survey-based age structure led to the best precision (Figs. 2 and 3, Additional file 1: Table S5, Tables S8-S9). However, when modelling mumps and parvovirus B19, in the three models used, the precision of the overall seroprevalence was found to be better using a uniform or population-based age structure (Figs. 2 and 4, Additional file 1: Table S6, Tables S10-S12). Finally, the precision for the estimated overall rubella seroprevalence was similar for the three different age structures (Fig. 2, Additional file 1: Table S7).

The precision of the estimated overall force of infection was better when using the survey-based age structure for VZV infection, in both models used (Fig. 3, Additional file 1: Tables S8-S9), and for parvovirus B19 infection under the MSIRWb-ext AW model, and using a uniform or population-based age structure for parvovirus B19 infection in the two other models used (Fig. 4, Additional file 1: Tables S10-S12).

For all the pathogens, as could be expected given the oversampling in children and adolescents in the survey-based age structure, the precision of the estimated seroprevalence by age group was better when using the survey-based age structure in the young age groups and the uniform or population-based age structure for the oldest age groups (Additional file 1: Tables S5-S12). The same pattern was observed for the force of infection of VZV and parvovirus B19 by age group (Additional file 1: Tables S8-S12).

In the exponentially damped model, the precision of R_0_ and the average age at infection was slightly better using the uniform or population-based age structure for parvovirus B19 while it was better using the survey-based age structure for VZV (Additional file 1: Tables S8 and S10). In the MSIRWb-ext AW model, the precision of R_0_, R_eff_, and the average age at infection of parvovirus B19 was slightly better using the survey-based age structure while that of the relative boosting factor (φ) was better using the uniform or population-based age structure (Additional file 1: Figure S4 and Table S12). However, the precision of this factor was poor, with large confidence intervals, and the average age at infection should be interpreted with caution given the bimodal force of infection.

### Sample size needed

To obtain a 2% precision around the overall seroprevalence estimate, the sample size needed would be around 1650 for mumps and parvovirus B19, while a lower number of samples would be sufficient for measles, VZV, and rubella; to obtain a 1% precision the sample size needed would be around 6600 for mumps and parvovirus B19, and 1650 for measles, VZV, and rubella (Figs. 2, 3, and 4; Additional file 1: Tables S5-S12). These results were quite consistent across age structures.

### Optimal allocation of a fixed sample size among age groups

For the overall seroprevalence of measles, mumps, or rubella, the optimal allocation (distribution over age groups) of a fixed number of samples would be a distribution with a high percentage of the data among age groups [19,31) and [31,65] years, for each sample size used (Additional file 1: Table S13-S15). Regarding the seroprevalence by age group, for measles, mumps and rubella, we have noticed some variations across the sample sizes; the optimal allocations were broadly uniform across the age groups.

**Fig. 5:**
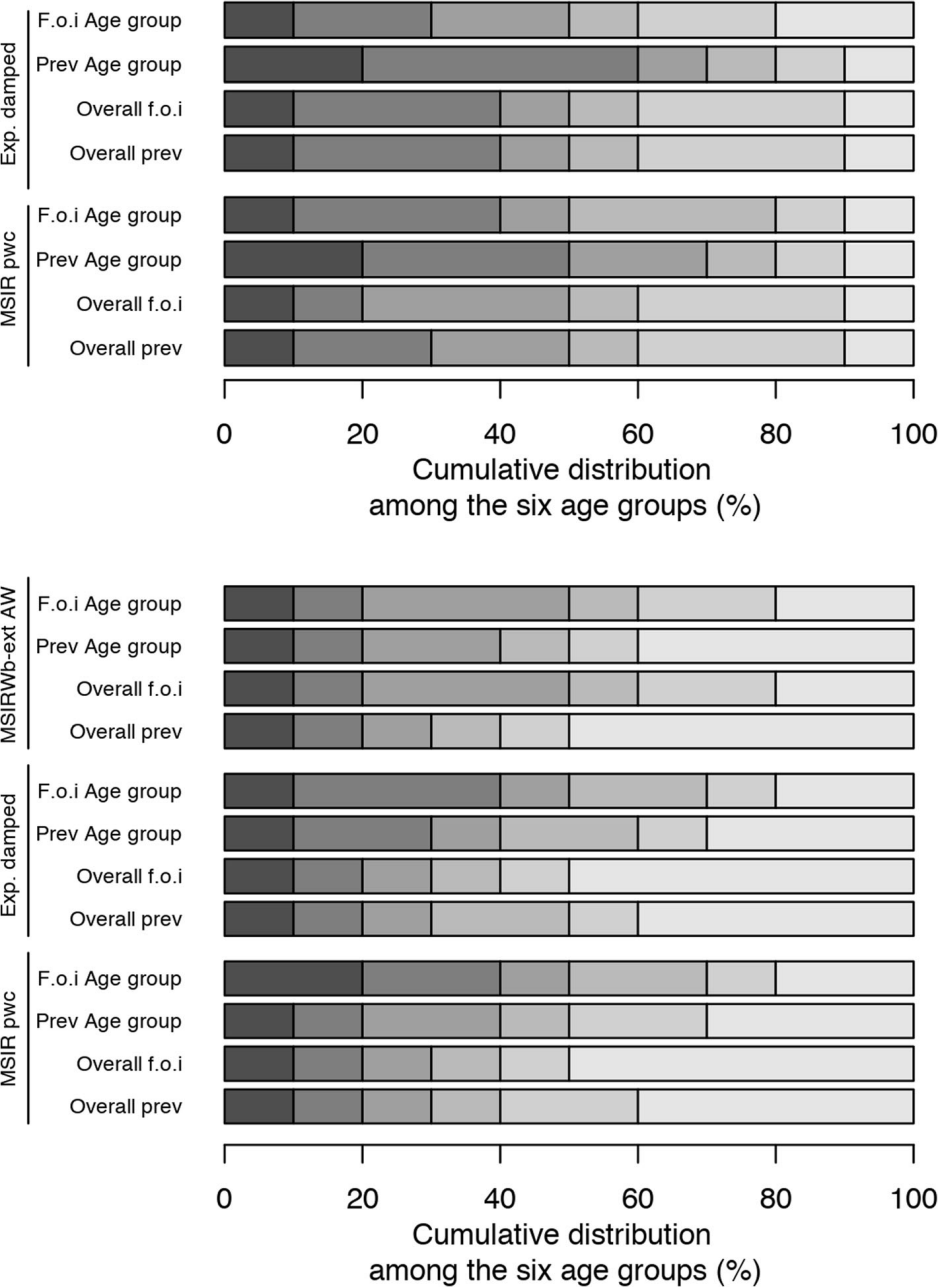
Optimal allocation (*N* = 3300) for various epidemiological parameters and by model (y-axis) among the six age groups (with lighter shades with increasing age group): [1,2), [2,6), [6,12), [12,19), [19,31), and [31,65] years, varicella-zoster virus (top) and parvovirus B19 (bottom) serological data. MSIR pcw: MSIR model with piecewise constant force of infection; Exp. damped: exponentially damped model; MSIRWb-ext AW: Maternally-derived immunity-Susceptible-Infectious-Recovered model allowing for age-specific waning of disease-acquired antibodies and boosting of low immunity; f.o.i: force of infection; Prev: prevalence

The optimal allocation for the overall VZV seroprevalence or force of infection estimates varied with sample size; the oldest two age groups would rather be favoured (Fig. 5 and Additional file 1: Tables S16-S17). The optimal allocation for the overall parvovirus B19 seroprevalence estimate would be a distribution with a high percentage of data in the oldest age group, for each model and sample size used (Fig. 5 and Additional file 1: Tables S18-S20). Regarding the overall force of infection of parvovirus B19, the optimal allocation would entail a distribution with high percentage among the oldest age group in the MSIR model with piecewise constant force of infection and exponentially damped model, while more equally distributed over the various age groups for the MSIRWb-ext AW model.

Regarding the seroprevalence or force of infection by age group for VZV and parvovirus B19, some variations between models and sizes were observed; the optimal allocations were broadly uniform across the age groups.

## Discussion

Considering sample size and optimal allocation is essential since efficient usage of resources is needed in the context of limited human or financial resources and/or time constraints for performing a serological survey. Since analytical formulas for complex models are not available, simulation-based analyses are a flexible alternative to address these considerations. In this paper, we proposed a simulation-based approach for sample size and age structure considerations, and optimal allocation of resources, in order to estimate key epidemiological parameters with acceptable levels of precision within the context of a single cross-sectional serological survey.

Our results showed that the best age structure to use in the sampling of a serological study as well as the optimal allocation distribution varied with the epidemiological parameters of interest. To our knowledge, only a few studies investigated, using mathematical or statistical models, the optimal allocation of a given number of samples over age groups to obtain good precision. Marschner [4] showed, using an example of measles infection, that a uniform age distribution should not be optimal to obtain a good joint precision of the force of infection.

For all the infections investigated, due to the oversampling of individuals under 20 years old in the serological survey purposefully, the precision of the estimated seroprevalence by age group was better with the survey-based age structure in the young age groups and the uniform or population age structure for the oldest age groups. Moreover, because of the formulas used to compute the basic or effective reproduction number and the average age at infection, the age structure best suited to estimate these parameters was related to that of the prevalence in the exponentially damped model and of the force of infection in the MSIRWb-ext AW model. In case the boosting rate is of interest, sufficiently sampling adults is essential. Anyway, the precision of this rate was poor as was also observed in previous analyses [16]. This could be explained by the complexity of the model used.

Our results showed that, to reach a given precision level around the overall seroprevalence estimate, the sample size needed would be higher for mumps and parvovirus B19 infections, compared to measles, VZV, and rubella infections. This may be explained by the fact that the prevalence levels across age groups were less variable for measles, VZV, and rubella, with a prevalence reaching relatively high values at young ages, compared to mumps and parvovirus B19.

An important finding was that the age-specific prevalence profile, and thus the age-specific force of infection profile, had an effect on the optimal age structure to use in a serological survey or the optimal allocation for estimating the overall seroprevalence. Indeed, the optimal age structure varied between VZV and parvovirus B19 infections, the seroprevalence increasing more sharply between ages 1 and 10 for VZV compared to parvovirus B19.

A main assumption was the existence of an endemic equilibrium for VZV and parvovirus B19 infections (i.e. the epidemic in a steady state). Under this assumption, the incidence might go through cyclical epidemics over time but oscillates around a stationary average value. However, although VZV or parvovirus B19 infections may undergo regular epidemic cycles, the serological survey took place on a relatively long time period (around 17 months), which would average these potential cycles. Moreover, this would have a limited impact on our results [25]. For parvovirus B19, although lifelong protection against infection upon recovery is questionable, this does not seem to be due to time heterogeneity since similar patterns were seen in other countries at different time points [18, 20, 21].

Our analyses could be extended to power analyses in the context of hypothesis testing. Indeed, data sets could be simulated assuming that an alternative hypothesis is true, then tested against the null hypothesis to calculate the proportion of simulated data sets in which the null hypothesis is rejected, thereby providing an estimate of the statistical power. Other possible extensions are related to non-endemic settings. An endemic equilibrium cannot be assumed for vaccine-preventable infections such as measles, mumps, and rubella for which a national immunisation programme is in place. In such settings, dynamical mathematical models allowing time considerations could be used to calculate the sample size needed for estimating time-varying parameters with acceptable precision levels or to perform power calculations to detect changes in parameter values over time, but this needs to be investigated. In particular, these analyses could make use of serial seroprevalence surveys (i.e., repeated collections of cross-sectional population-representative serological samples) [9]. Finally, our analyses could also be extended to more complex models, for example transmission models including maternal antibody waning in newborns or incorporating the presence of individual heterogeneities [26, 27].

Our analyses had some limitations. First, the number of age groups to optimally allocate a given number of samples had to be limited to avoid a huge number of combinations. Here, six age groups were used leading to 126 distributions. Alternative age groups of interest or a predetermined age distribution (e.g., derived from previous surveys or population-based) can be used. Moreover, the optimal allocation will depend on the rule used to calculate the joint precision. Here, we used the sum of the age-specific precisions. Alternative rules could be considered such as the sum of the relative precisions. However, favouring very small values could result in a very large sample size or be of less interest (e.g., if force of infection in older age groups is known to be small).

Second, the use of measurements of antibody levels based on diagnostic tests relies on the assumption of a perfect test (i.e., both sensitive and specific). In lack of which, discrepancies between the seroprevalence and the disease prevalence are observed in the presence of misclassification, which would alter the estimates of the overall and age-specific prevalence, even more if sensitivity and specificity vary with age [28]. The estimate of the seroprevalence can be corrected if estimates of the sensitivity and specificity of the test(s) applied are available [29]. Alternatively, mixture modelling of continuous antibody titres can be used; however the combination of this technique with mathematical models needs further investigations [2, 30–32]. In the current work, considering misclassifications negligible appeared reasonable.

Finally, like other standard methods, the approach presented here would require prior knowledge about parameter values: e.g., (sero)prevalence or force of infection by age (group) to simulate data. However, sensitivity analyses may be performed to assess how this prior knowledge affects the sample size needed or optimal allocation and would inform about the minimum sample size needed. Here, data from 2002 were used to illustrate our approach but, although the endemic equilibrium assumption for parvovirus B19 and VZV is believed to be reasonable, more recent estimates should be used to plan future studies.

In any case, the choice of sampling design or modelling approach should be adapted to prior knowledge about the infection and the precision of estimates (overall or age-specific) should be considered in the context of the study goals and the anticipated implications for infection control measures or vaccine programs.

## Conclusions

The main conclusions from the presented analyses are that attention should be given to the age-based sampling structure when estimating key epidemiological parameters with acceptable levels of precision within the context of a single cross-sectional serological survey, and that simulation-based sample size calculations in combination with mathematical modelling can be utilised for choosing the optimal allocation of a given number of samples over various age groups.

## Additional file

Additional file 1: Supplementary material. (PDF 1693 kb)

## Abbreviations

CI: confidence interval
ESEN: European Sero-Epidemiology Network
GLM: generalized linear model
MSIR model: Maternally-derived immunity-Susceptible-Infectious-Recovered model
MSIRWb-ext AW model: MSIR model allowing for age-specific waning of disease-acquired antibodies and boosting of low immunity
ODE: ordinary differential equation
SIR model: Susceptible-Infectious-Recovered model
VZV: varicella-zoster virus
