# Supplementary material for: Sample size calculation for estimating key epidemiological parameters using serological data and mathematical modelling
Source: BMC Med Res Methodol. 2019 Mar 7;19:51. doi: 10.1186/s12874-019-0692-1 (PMC6407263; doi:10.1186/s12874-019-0692-1)
Supplement: Supplementary file 1 — Supplementary material. (PDF 1693 kb) [file 12874_2019_692_MOESM1_ESM.pdf]

Sample size calculation for estimating key epidemiological parameters using serological data and mathematical modelling

Stéphanie Blaizot, Sereina A. Herzog, Steven Abrams, Heidi Theeten, Amber Litzroth, Niel Hens

Supplementary material

Contents

Age structures ... 2

MSIRW model with boosting and age-specific waning (MSIRWb-ext AW) ... 3

Calculations of the key epidemiological parameters ... 4

Estimates of key epidemiological parameters ... 5

Precision values ... 7

Optimal distributions ... 18

References ... 25

Age structures

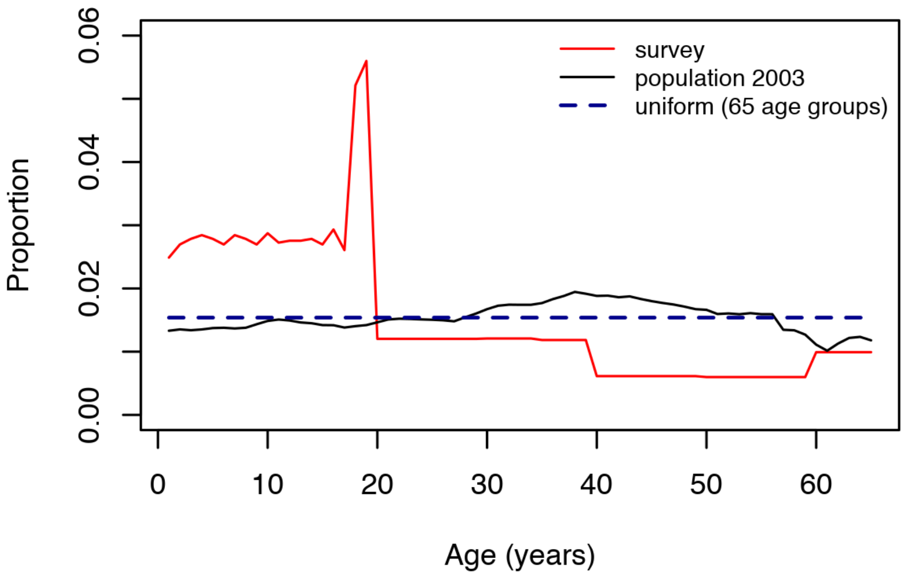

Figure S1. Comparison of the three age distributions.

"survey" (red line) refers to the age structure derived from the serological survey data (all individuals included, aged from 1 to 65 years old); "population 2003" (black line) refers to the age structure of the Belgian population in 2003; "uniform" (blue dashed line) refers to a uniform age structure based on 65 age groups (1-year interval).

Table S1. Distribution of the population among age groups by age structure

	**Age groups**					
	1 year	2-5 years	6-11 years	12-18 years	19-30 years	31-65 years
**Population**	1.1%	4.5%	7.2%	8.4%	15.3%	63.5%
**Uniform**	1.5%	6.2%	9.2%	10.8%	18.5%	53.8%
**Serological survey**						
Measles	2.3%	11.1%	16.5%	20.9%	18.5%	30.6%
Mumps	2.5%	11.3%	16.3%	21.6%	18.6%	29.7%
Rubella	2.5%	11.2%	16.2%	21.4%	18.8%	29.9%
Parvovirus B19	1.9%	9.3%	15.4%	21.7%	19.9%	31.8%
VZV	2.6%	11.3%	16.6%	21.7%	18.2%	29.8%

The "serological survey" structure refers to the structure of the pathogen-specific measures available. VZV: varicella-zoster virus.

MSIRW model with boosting and age-specific waning (MSIRWb-ext AW)

In Goeyvaerts *et al*. (2011), different compartmental models were considered to infer on basic immunological processes for parvovirus B19 (Goeyvaerts et al. 2011). In the MSIRWb-ext AW model, they considered waning of disease-acquired antibodies. Individuals move at a rate $\varepsilon(a)$ from a high immunity state R to a low immunity state W in which they are still protected from infection however categorized as being seronegative, that is, with antibody levels falling below the serostatus threshold (see Figure S2). In addition, they assumed that low immunity can be boosted by exposure to infectious individuals. The boosting rate and the force of infection are proportional with a proportionality constant φ, such that the rate at which individuals move back from W to R equals $\varphi \cdot \lambda(a)$. By solving the corresponding set of differential equations, one finds that the fraction in state S equals:

$$s(a) = \exp\left(-\int_A^a \lambda(u)\,\mathrm{d}u\right) \text{if } a > A.$$

Approximating $r(a)$ by $1 - s(a) - w(a)$, $\forall a > A$, assuming $i(a)$ is small relative to $s(a)$ and $w(a)$, the following expression is obtained for the proportion seropositives (if $a > A$):

$$r(a) = \int_A^a \left\{(1-\varphi)\,\lambda(u)\exp\left(-\int_A^u \lambda(v)\mathrm{d}v\right) + \varphi\lambda(u)\right\} \exp\left(-\int_u^a \{\varphi\lambda(v) + \varepsilon(v)\}\,\mathrm{d}v\right)\mathrm{d}u$$

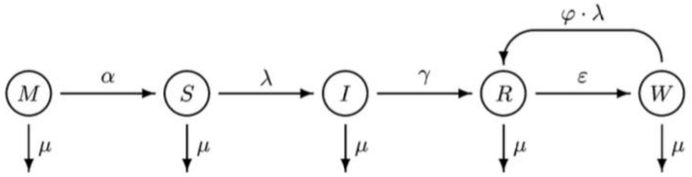

Figure S2. Illustration of the MSIRWb-ext compartmental model.

The estimation method assumes endemic and demographic equilibrium. The transmission rates $\beta(a,a')$ are assumed to be directly proportional to age-specific rates of making social contact, $c(a,a')$, with a disease-specific proportionality factor q ("constant proportionality assumption"): $\beta(a,a') = q \cdot c(a,a')$. The contact rates are estimated from the POLYMOD contact survey using a non-parametric model (Goeyvaerts et al. 2010). Since the above integral equation has no closed-form solution, we turn to discrete age classes to estimate the parameters. Through an iterative procedure, the Bernoulli log-likelihood for the serological data is maximized. The waning rate is modelled as a piecewise constant function with a cut-off point at predetermined age H: $\varepsilon(a) = \varepsilon_1$, if $a \in (A, H)$, and $\varepsilon(a) = \varepsilon_2$, if $a \geq H$. Comparing the overall likelihood for different values of H (from 5 to 50 years in 5 years steps) led to the choice of $H = 35$ years.

Calculations of the key epidemiological parameters

- Calculation of the overall prevalence: $\hat{p}_{overall} = \frac{\sum_a \hat{p}_a \times f_a}{\sum_a f_a}$ where $\hat{p}_a$ is the estimated age-specific prevalence and f_a the proportion of individuals aged a from the population distribution.
- Calculation of the overall force of infection: $\hat{\lambda}_{overall} = \frac{\sum_a \hat{\lambda}_a \times f_a \times (1-\hat{p}_a)}{\sum_a f_a \times (1-\hat{p}_a)}$ where $\hat{\lambda}_a$ is the estimated age-specific force of infection, $\hat{p}_a$ is the estimated age-specific prevalence and f_a the proportion of individuals aged *a* from the population distribution.
- Calculation of R_0:
  - Exponentially damped model: $1 + \frac{L}{A_{inf}}$
  - MSIRWb-ext AW model (Goeyvaerts et al. 2011): dominant eigenvalue of $G = \frac{ND}{L}\left(a_{[i+1]} - a_{[i]}\right)\beta_{ij}$ where β_{ij} denotes the per capita rate at which an individual of age class *j* makes an effective contact with a person of age class *i* per year.
- Calculation of the mean age at infection:
  - Exponentially damped model: $A_{inf} = \int_0^U [1 - \pi(a)]da + [1 - \pi(U)] \times (L - U)$ in case that the data are observed up to a certain age U ($U \leq L$)
  - MSIRWb-ext AW model (Goeyvaerts et al. 2011): $A_{inf} = \frac{\int_0^\infty a\lambda(a)s(a)N(a)/N(0)da}{n_{SI}}$ with $n_{SI} = \int_0^\infty \lambda(a)s(a)N(a)/N(0)da$ the average number of infections per person during their lifetime
- Calculation of R_{eff} (MSIRWb-ext AW model, (Santermans et al. 2015)): dominant eigenvalue of $G \times s(a)$

Estimates of key epidemiological parameters

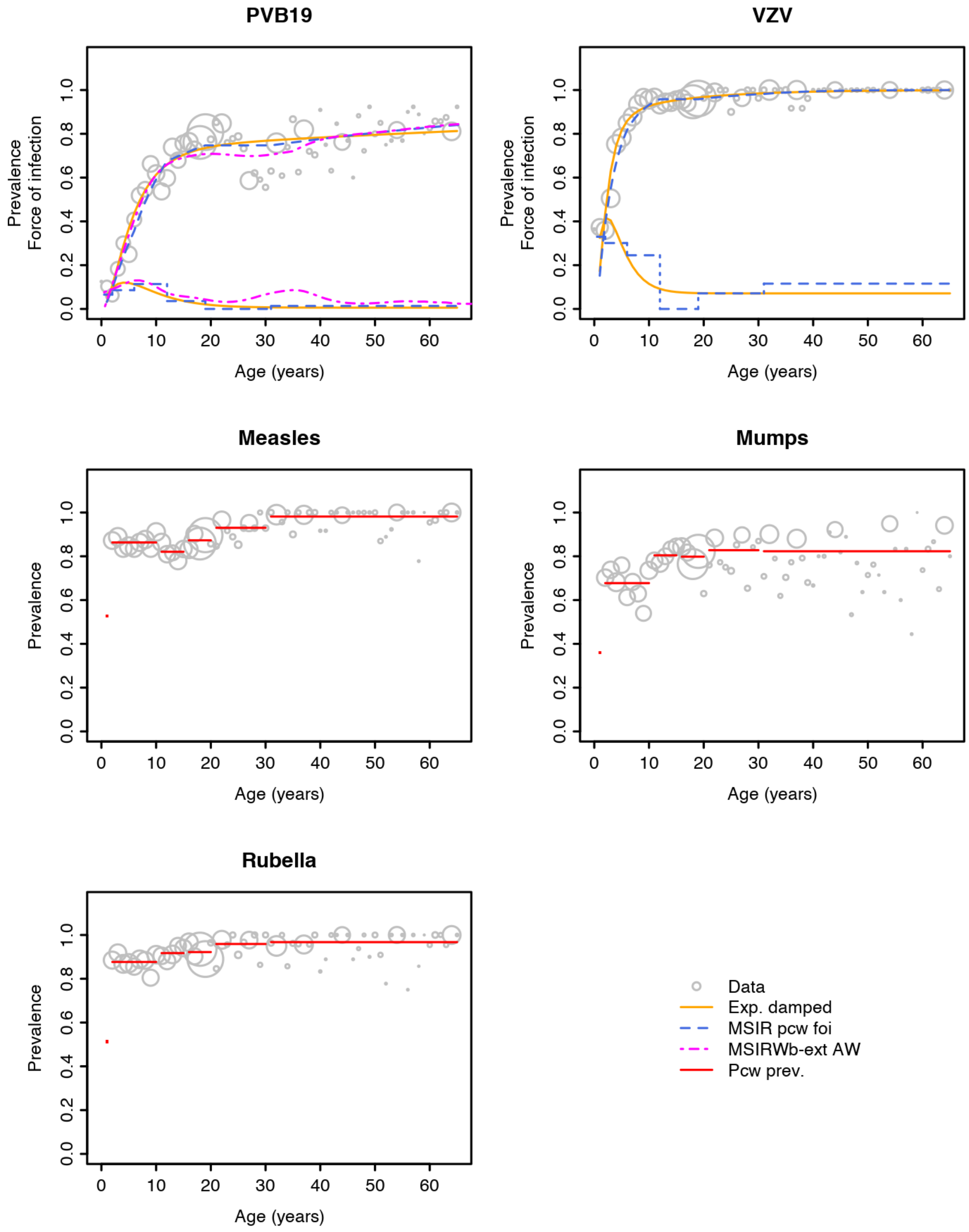

Figure S3. Estimated prevalence and force of infection for each of the pathogens.

Points are the observed data with circles proportional to sample size. PVB19: parvovirus B19; VZV: varicella-zoster virus; Exp. damped: exponentially damped model; MSIR – pwc foi: MSIR model with piecewise constant force of infection; MSIRWb-ext AW: MSIR model with boosting and age-specific waning; Pcw. prev: logistic model with piecewise constant prevalence.

Table S2. Estimates of the overall prevalence and prevalence by age groups by model and infection

	Prevalence						
	[1,2) yrs	[2,6) yrs	[6,12) yrs	[12,19) yrs	[19,31) yrs	[31,65] yrs	Overall
**MSIR with piecewise constant force of infection**							
VZV	18.3%	64.8%	91.0%	95.7%	97.1%	99.5%	95.0%
B19	3.8%	23.3%	53.8%	70.9%	74.8%	79.5%	71.5%
**Exponentially damped model**							
VZV	17.3%	65.7%	90.6%	95.3%	97.6%	99.4%	95.0%
B19	3.3%	24.1%	55.4%	69.9%	75.5%	79.1%	71.5%
**MSIRWb-ext AW** B19	4.1%	24.2%	56.7%	70.1%	70.4%	78.6%	70.5%
**Logistic model**							
Measles	52.7%	86.3%	85.6%	84.3%	92.1%	98.2%	93.3%
Mumps	36.0%	67.7%	70.0%	80.2%	82.3%	82.3%	79.6%
Rubella	51.2%	87.7%	88.4%	91.9%	95.3%	96.7%	94.2%

All models are applied to data with integer age values.

Table S3. Estimates of the overall force of infection and force of infection by age groups by model and infection

	Force of infection						
	[1,2) yrs	[2,6) yrs	[6,12) yrs	[12,19) yrs	[19,31) yrs	[31,65] yrs	Overall
**MSIR with piecewise constant force of infection**							
VZV	0.404	0.337	0.200	0.000	0.076	0.113	0.262
B19	0.077	0.104	0.100	0.035	0.000	0.014	0.042
**Exponentially damped model**							
VZV	0.325	0.385	0.168	0.081	0.071	0.071	0.260
B19	0.061	0.111	0.091	0.039	0.012	0.006	0.040
**MSIRWb-ext AW** B19	0.090	0.114	0.124	0.060	0.045	0.052	0.071

All models are applied to data with integer age values.

Table S4. Estimates of R_0, average age at infection, and R_{eff}, by model and infection

	R_0	Average age at infection	R_{eff}
**Exponentially damped model**			
VZV	22.92	3.6	-
B19	4.65	21.6	-
**MSIRWb-ext AW** B19	3.94	10.7	1.06

All models are applied to data with integer age values.

Precision values

In Tables S5-12, the following abbreviations are used:

survey: age structure derived from the serological survey data; uniform: uniform age structure; population: age structure of the Belgian population; OverallPrev: overall seroprevalence; Prev1 to Prev6: prevalence by age group ([1,2), [2,6), [6,12), [12,19), [19,31), and [31,65] years); OverallFoi: overall force of infection; Foi1 to Foi6: force of infection by age group ([1,2), [2,6), [6,12), [12,19), [19,31), and [31,65] years); R_0: basic reproduction number; R_{eff}: effective reproduction number; ASI: average age at infection.

Table S5. Measles serological data – Logistic model with piecewise constant prevalence

Size	Structure	OverallPrev	Prev1	Prev2	Prev3	Prev4	Prev5	Prev6
1650	survey	0.0106	0.1579	0.0330	0.0292	0.0372	0.0289	0.0119
3300	survey	0.0082	0.1104	0.0214	0.0193	0.0243	0.0214	0.0084
6600	survey	0.0054	0.0796	0.0160	0.0143	0.0166	0.0153	0.0053
9900	survey	0.0041	0.0628	0.0136	0.0115	0.0142	0.0125	0.0047
13200	survey	0.0038	0.0552	0.0111	0.0100	0.0125	0.0110	0.0044
19800	survey	0.0031	0.0478	0.0096	0.0081	0.0091	0.0085	0.0033
1650	uniform	0.0110	0.2200	0.0434	0.0395	0.0463	0.0270	0.0083
3300	uniform	0.0084	0.1353	0.0300	0.0274	0.0335	0.0197	0.0066
6600	uniform	0.0055	0.0990	0.0215	0.0180	0.0226	0.0145	0.0042
9900	uniform	0.0047	0.0789	0.0174	0.0153	0.0193	0.0118	0.0032
13200	uniform	0.0038	0.0665	0.0153	0.0134	0.0169	0.0099	0.0032
19800	uniform	0.0032	0.0559	0.0130	0.0110	0.0134	0.0084	0.0027
1650	population	0.0109	0.2045	0.0447	0.0425	0.0468	0.0273	0.0086
3300	population	0.0084	0.1423	0.0336	0.0287	0.0348	0.0196	0.0059
6600	population	0.0056	0.1023	0.0219	0.0190	0.0234	0.0147	0.0042
9900	population	0.0048	0.0833	0.0187	0.0160	0.0198	0.0119	0.0032
13200	population	0.0039	0.0710	0.0161	0.0144	0.0156	0.0102	0.0031
19800	population	0.0031	0.0625	0.0141	0.0116	0.0146	0.0081	0.0025

Table S6. Mumps serological data – Logistic model with piecewise constant prevalence

Size	Structure	OverallPrev	Prev1	Prev2	Prev3	Prev4	Prev5	Prev6
1650	survey	0.0207	0.1463	0.0439	0.0364	0.0367	0.0442	0.0316
3300	survey	0.0153	0.0964	0.0320	0.0261	0.0251	0.0304	0.0233
6600	survey	0.0112	0.0693	0.0212	0.0182	0.0171	0.0232	0.0164
9900	survey	0.0093	0.0615	0.0184	0.0160	0.0149	0.0176	0.0138
13200	survey	0.0075	0.0537	0.0161	0.0138	0.0129	0.0167	0.0111
19800	survey	0.0065	0.0408	0.0133	0.0110	0.0099	0.0132	0.0104
1650	uniform	0.0187	0.1800	0.0589	0.0527	0.0515	0.0443	0.0240
3300	uniform	0.0136	0.1300	0.0406	0.0350	0.0371	0.0284	0.0181
6600	uniform	0.0092	0.0941	0.0303	0.0248	0.0243	0.0211	0.0132
9900	uniform	0.0078	0.0724	0.0243	0.0198	0.0210	0.0177	0.0106
13200	uniform	0.0065	0.0640	0.0194	0.0169	0.0178	0.0157	0.0083
19800	uniform	0.0054	0.0551	0.0181	0.0154	0.0156	0.0121	0.0076
1650	population	0.0182	0.2045	0.0617	0.0538	0.0508	0.0427	0.0226
3300	population	0.0140	0.1364	0.0439	0.0367	0.0358	0.0278	0.0171
6600	population	0.0093	0.0996	0.0313	0.0260	0.0263	0.0211	0.0125
9900	population	0.0078	0.0758	0.0244	0.0205	0.0215	0.0168	0.0107
13200	population	0.0063	0.0668	0.0211	0.0181	0.0171	0.0150	0.0081
19800	population	0.0054	0.0606	0.0189	0.0158	0.0149	0.0117	0.0075

Table S7. Rubella serological data – Logistic model with piecewise constant prevalence

Size	Structure	OverallPrev	Prev1	Prev2	Prev3	Prev4	Prev5	Prev6
1650	survey	0.0111	0.1548	0.0336	0.0279	0.0251	0.0220	0.0152
3300	survey	0.0085	0.1084	0.0215	0.0183	0.0174	0.0171	0.0120
6600	survey	0.0057	0.0723	0.0161	0.0139	0.0121	0.0123	0.0076
9900	survey	0.0046	0.0622	0.0133	0.0112	0.0110	0.0100	0.0061
13200	survey	0.0037	0.0557	0.0113	0.0095	0.0090	0.0083	0.0054
19800	survey	0.0033	0.0462	0.0091	0.0078	0.0072	0.0073	0.0043
1650	uniform	0.0105	0.2000	0.0423	0.0370	0.0345	0.0215	0.0120
3300	uniform	0.0074	0.1300	0.0312	0.0256	0.0240	0.0154	0.0080
6600	uniform	0.0055	0.0967	0.0204	0.0177	0.0168	0.0118	0.0057
9900	uniform	0.0046	0.0822	0.0170	0.0146	0.0146	0.0088	0.0043
13200	uniform	0.0037	0.0678	0.0151	0.0131	0.0117	0.0075	0.0038
19800	uniform	0.0032	0.0593	0.0122	0.0103	0.0100	0.0062	0.0034
1650	population	0.0105	0.2045	0.0411	0.0376	0.0346	0.0231	0.0113
3300	population	0.0075	0.1477	0.0329	0.0269	0.0241	0.0152	0.0077
6600	population	0.0053	0.1026	0.0213	0.0184	0.0175	0.0114	0.0058
9900	population	0.0045	0.0871	0.0177	0.0146	0.0146	0.0089	0.0043
13200	population	0.0037	0.0739	0.0158	0.0136	0.0122	0.0076	0.0036
19800	population	0.0033	0.0625	0.0137	0.0113	0.0106	0.0065	0.0033

Table S8. VZV serological data – Exponentially damped model

Size	Structure	OverallPrev	Prev1	Prev2	Prev3	Prev4	Prev5	Prev6
1650	survey	0.0071	0.0520	0.0591	0.0250	0.0142	0.0102	0.0056
3300	survey	0.0049	0.0333	0.0386	0.0160	0.0103	0.0069	0.0043
6600	survey	0.0037	0.0240	0.0285	0.0126	0.0075	0.0048	0.0028
9900	survey	0.0026	0.0220	0.0242	0.0103	0.0059	0.0031	0.0018
13200	survey	0.0023	0.0184	0.0206	0.0087	0.0052	0.0032	0.0020
19800	survey	0.0021	0.0142	0.0156	0.0068	0.0045	0.0027	0.0016
1650	uniform	0.0085	0.0716	0.0785	0.0320	0.0182	0.0091	0.0041
3300	uniform	0.0060	0.0493	0.0585	0.0212	0.0131	0.0067	0.0032
6600	uniform	0.0040	0.0328	0.0386	0.0152	0.0095	0.0047	0.0021
9900	uniform	0.0038	0.0268	0.0307	0.0127	0.0077	0.0038	0.0018
13200	uniform	0.0031	0.0234	0.0280	0.0114	0.0064	0.0032	0.0015
19800	uniform	0.0023	0.0195	0.0227	0.0084	0.0056	0.0026	0.0013
1650	population	0.0090	0.0779	0.0881	0.0323	0.0187	0.0091	0.0041
3300	population	0.0057	0.0508	0.0572	0.0226	0.0131	0.0065	0.0029
6600	population	0.0042	0.0352	0.0412	0.0154	0.0101	0.0047	0.0022
9900	population	0.0035	0.0284	0.0325	0.0128	0.0082	0.0039	0.0017
13200	population	0.0029	0.0255	0.0282	0.0115	0.0069	0.0032	0.0014
19800	population	0.0025	0.0205	0.0252	0.0091	0.0056	0.0027	0.0012

Size	Structure	OverallFoi	Foi1	Foi2	Foi3	Foi4	Foi5	Foi6
1650	survey	0.0353	0.0906	0.0518	0.0473	0.0359	0.0434	0.0441
3300	survey	0.0239	0.0603	0.0326	0.0350	0.0234	0.0288	0.0291
6600	survey	0.0179	0.0434	0.0236	0.0244	0.0146	0.0187	0.0189
9900	survey	0.0129	0.0395	0.0185	0.0230	0.0101	0.0138	0.0141
13200	survey	0.0114	0.0325	0.0171	0.0182	0.0115	0.0146	0.0148
19800	survey	0.0100	0.0252	0.0120	0.0153	0.0082	0.0108	0.0109
1650	uniform	0.0416	0.1268	0.0658	0.0616	0.0280	0.0330	0.0338
3300	uniform	0.0294	0.0891	0.0439	0.0470	0.0192	0.0245	0.0251
6600	uniform	0.0195	0.0601	0.0299	0.0341	0.0134	0.0163	0.0168
9900	uniform	0.0176	0.0474	0.0257	0.0268	0.0102	0.0133	0.0137
13200	uniform	0.0150	0.0425	0.0220	0.0227	0.0086	0.0115	0.0118
19800	uniform	0.0113	0.0352	0.0174	0.0198	0.0070	0.0089	0.0092
1650	population	0.0433	0.1410	0.0689	0.0659	0.0273	0.0339	0.0351
3300	population	0.0279	0.0907	0.0452	0.0487	0.0184	0.0251	0.0257
6600	population	0.0201	0.0629	0.0309	0.0362	0.0133	0.0162	0.0166
9900	population	0.0172	0.0509	0.0249	0.0282	0.0098	0.0132	0.0135
13200	population	0.0143	0.0453	0.0241	0.0253	0.0086	0.0113	0.0115
19800	population	0.0118	0.0371	0.0186	0.0211	0.0072	0.0090	0.0092

Size	Structure	R_0	ASI
1650	survey	3.0545	0.4995
3300	survey	2.1016	0.3472
6600	survey	1.5552	0.2533
9900	survey	1.1384	0.1840
13200	survey	0.9811	0.1599
19800	survey	0.8942	0.1464
1650	uniform	3.7604	0.5920
3300	uniform	2.5140	0.4081
6600	uniform	1.7357	0.2816
9900	uniform	1.6336	0.2674
13200	uniform	1.3314	0.2190
19800	uniform	1.0348	0.1679
1650	population	3.9463	0.6310
3300	population	2.4735	0.4046
6600	population	1.8329	0.3006
9900	population	1.5411	0.2524
13200	population	1.2795	0.2096
19800	population	1.0671	0.1741

Table S9. VZV serological data – MSIR piecewise constant force of infection

Size	Structure	OverallPrev	Prev1	Prev2	Prev3	Prev4	Prev5	Prev6
1650	survey	0.0073	0.0575	0.0610	0.0251	0.0162	0.0136	0.0046
3300	survey	0.0049	0.0359	0.0402	0.0177	0.0115	0.0101	0.0038
6600	survey	0.0038	0.0279	0.0292	0.0128	0.0079	0.0071	0.0028
9900	survey	0.0030	0.0221	0.0236	0.0103	0.0068	0.0054	0.0023
13200	survey	0.0024	0.0210	0.0204	0.0090	0.0057	0.0047	0.0020
19800	survey	0.0020	0.0158	0.0159	0.0073	0.0050	0.0040	0.0016
1650	uniform	0.0087	0.0798	0.0764	0.0352	0.0226	0.0137	0.0038
3300	uniform	0.0060	0.0543	0.0557	0.0254	0.0158	0.0102	0.0029
6600	uniform	0.0042	0.0342	0.0387	0.0172	0.0111	0.0072	0.0022
9900	uniform	0.0036	0.0314	0.0310	0.0137	0.0097	0.0055	0.0017
13200	uniform	0.0032	0.0259	0.0287	0.0119	0.0081	0.0050	0.0014
19800	uniform	0.0023	0.0217	0.0221	0.0097	0.0062	0.0038	0.0012
1650	population	0.0089	0.0842	0.0880	0.0360	0.0222	0.0140	0.0037
3300	population	0.0061	0.0564	0.0587	0.0249	0.0167	0.0101	0.0028
6600	population	0.0043	0.0373	0.0417	0.0173	0.0116	0.0071	0.0021
9900	population	0.0036	0.0305	0.0339	0.0142	0.0098	0.0056	0.0016
13200	population	0.0031	0.0292	0.0305	0.0131	0.0080	0.0049	0.0014
19800	population	0.0025	0.0219	0.0241	0.0104	0.0065	0.0039	0.0012

Size	Structure	OverallFoi	Foi1	Foi2	Foi3	Foi4	Foi5	Foi6
1650	survey	0.0383	0.1416	0.1307	0.1137	0.0711	0.0894	0.3651
3300	survey	0.0263	0.0879	0.0910	0.0853	0.0461	0.0774	0.2816
6600	survey	0.0200	0.0683	0.0560	0.0573	0.0389	0.0570	0.1015
9900	survey	0.0152	0.0542	0.0502	0.0470	0.0285	0.0482	0.0778
13200	survey	0.0134	0.0514	0.0439	0.0417	0.0253	0.0379	0.0610
19800	survey	0.0110	0.0386	0.0365	0.0316	0.0207	0.0294	0.0480
1650	uniform	0.0461	0.1983	0.1555	0.1422	0.0919	0.0892	0.2131
3300	uniform	0.0340	0.1339	0.1216	0.1100	0.0661	0.0717	0.1013
6600	uniform	0.0240	0.0837	0.0834	0.0807	0.0454	0.0584	0.0679
9900	uniform	0.0189	0.0772	0.0660	0.0626	0.0420	0.0463	0.0496
13200	uniform	0.0159	0.0633	0.0516	0.0536	0.0361	0.0402	0.0430
19800	uniform	0.0125	0.0531	0.0471	0.0430	0.0266	0.0317	0.0364
1650	population	0.0453	0.2088	0.1763	0.1616	0.0937	0.0819	0.2257
3300	population	0.0336	0.1386	0.1282	0.1117	0.0661	0.0671	0.0989
6600	population	0.0233	0.0915	0.0869	0.0866	0.0446	0.0593	0.0645
9900	population	0.0190	0.0748	0.0691	0.0652	0.0405	0.0445	0.0540
13200	population	0.0158	0.0715	0.0587	0.0567	0.0379	0.0383	0.0439
19800	population	0.0131	0.0537	0.0486	0.0471	0.0255	0.0315	0.0331

Table S10. Parvovirus B19 serological data – Exponentially damped model

Size	Structure	OverallPrev	Prev1	Prev2	Prev3	Prev4	Prev5	Prev6
1650	survey	0.0215	0.0094	0.0415	0.0391	0.0305	0.0285	0.0294
3300	survey	0.0148	0.0074	0.0321	0.0298	0.0213	0.0202	0.0208
6600	survey	0.0106	0.0049	0.0213	0.0203	0.0156	0.0148	0.0148
9900	survey	0.0092	0.0039	0.0175	0.0163	0.0115	0.0120	0.0138
13200	survey	0.0073	0.0033	0.0144	0.0135	0.0099	0.0105	0.0109
19800	survey	0.0071	0.0029	0.0132	0.0117	0.0087	0.0085	0.0100
1650	uniform	0.0199	0.0126	0.0552	0.0511	0.0376	0.0321	0.0226
3300	uniform	0.0152	0.0086	0.0392	0.0407	0.0268	0.0218	0.0166
6600	uniform	0.0097	0.0062	0.0278	0.0281	0.0175	0.0160	0.0120
9900	uniform	0.0082	0.0048	0.0219	0.0203	0.0150	0.0135	0.0098
13200	uniform	0.0069	0.0042	0.0190	0.0184	0.0120	0.0111	0.0079
19800	uniform	0.0055	0.0033	0.0149	0.0145	0.0094	0.0092	0.0074
1650	population	0.0192	0.0135	0.0570	0.0536	0.0366	0.0300	0.0215
3300	population	0.0142	0.0090	0.0402	0.0416	0.0277	0.0223	0.0173
6600	population	0.0097	0.0065	0.0292	0.0269	0.0176	0.0159	0.0117
9900	population	0.0079	0.0050	0.0233	0.0224	0.0152	0.0130	0.0101
13200	population	0.0071	0.0043	0.0201	0.0200	0.0128	0.0115	0.0081
19800	population	0.0059	0.0038	0.0174	0.0162	0.0096	0.0091	0.0076

Size	Structure	OverallFoi	Foi1	Foi2	Foi3	Foi4	Foi5	Foi6
1650	survey	0.0050	0.0167	0.0174	0.0117	0.0117	0.0054	0.0064
3300	survey	0.0036	0.0131	0.0130	0.0084	0.0091	0.0040	0.0056
6600	survey	0.0025	0.0087	0.0089	0.0057	0.0065	0.0028	0.0038
9900	survey	0.0023	0.0070	0.0071	0.0049	0.0053	0.0024	0.0036
13200	survey	0.0020	0.0058	0.0059	0.0044	0.0045	0.0020	0.0031
19800	survey	0.0018	0.0053	0.0054	0.0034	0.0039	0.0017	0.0025
1650	uniform	0.0037	0.0223	0.0223	0.0131	0.0142	0.0055	0.0065
3300	uniform	0.0030	0.0154	0.0168	0.0093	0.0098	0.0039	0.0051
6600	uniform	0.0019	0.0110	0.0117	0.0065	0.0072	0.0027	0.0038
9900	uniform	0.0018	0.0086	0.0090	0.0055	0.0062	0.0022	0.0033
13200	uniform	0.0014	0.0075	0.0079	0.0045	0.0052	0.0019	0.0027
19800	uniform	0.0013	0.0059	0.0062	0.0036	0.0041	0.0016	0.0021
1650	population	0.0038	0.0241	0.0240	0.0141	0.0138	0.0055	0.0067
3300	population	0.0029	0.0161	0.0174	0.0094	0.0101	0.0040	0.0054
6600	population	0.0021	0.0118	0.0120	0.0069	0.0077	0.0027	0.0040
9900	population	0.0019	0.0090	0.0097	0.0055	0.0060	0.0022	0.0034
13200	population	0.0015	0.0077	0.0083	0.0045	0.0053	0.0020	0.0029
19800	population	0.0013	0.0068	0.0071	0.0036	0.0045	0.0017	0.0023

Size	Structure	R_0	ASI
1650	survey	0.3125	1.8265
3300	survey	0.2283	1.3602
6600	survey	0.1537	0.9046
9900	survey	0.1415	0.8382
13200	survey	0.1198	0.7077
19800	survey	0.1081	0.6394
1650	uniform	0.2661	1.5597
3300	uniform	0.2004	1.1785
6600	uniform	0.1320	0.7797
9900	uniform	0.1140	0.6742
13200	uniform	0.0927	0.5481
19800	uniform	0.0817	0.4841
1650	population	0.2550	1.4957
3300	population	0.1932	1.1408
6600	population	0.1361	0.8059
9900	population	0.1161	0.6863
13200	population	0.0951	0.5629
19800	population	0.0843	0.4997

Table S11. Parvovirus B19 serological data – MSIR piecewise constant force of infection

Size	Structure	OverallPrev	Prev1	Prev2	Prev3	Prev4	Prev5	Prev6
1650	survey	0.0205	0.0277	0.0483	0.0498	0.0378	0.0341	0.0284
3300	survey	0.0150	0.0203	0.0383	0.0350	0.0246	0.0235	0.0199
6600	survey	0.0102	0.0149	0.0264	0.0241	0.0195	0.0178	0.0147
9900	survey	0.0092	0.0118	0.0204	0.0199	0.0153	0.0140	0.0130
13200	survey	0.0074	0.0099	0.0183	0.0169	0.0130	0.0118	0.0105
19800	survey	0.0074	0.0080	0.0156	0.0142	0.0099	0.0098	0.0099
1650	uniform	0.0199	0.0331	0.0633	0.0569	0.0458	0.0371	0.0225
3300	uniform	0.0144	0.0232	0.0470	0.0471	0.0370	0.0272	0.0161
6600	uniform	0.0100	0.0163	0.0322	0.0304	0.0238	0.0183	0.0128
9900	uniform	0.0079	0.0140	0.0261	0.0248	0.0200	0.0155	0.0099
13200	uniform	0.0067	0.0121	0.0235	0.0225	0.0183	0.0131	0.0081
19800	uniform	0.0057	0.0095	0.0187	0.0183	0.0138	0.0103	0.0075
1650	population	0.0196	0.0387	0.0683	0.0614	0.0473	0.0356	0.0223
3300	population	0.0141	0.0253	0.0473	0.0468	0.0385	0.0266	0.0159
6600	population	0.0097	0.0181	0.0343	0.0330	0.0245	0.0181	0.0120
9900	population	0.0079	0.0148	0.0272	0.0256	0.0196	0.0150	0.0098
13200	population	0.0072	0.0128	0.0241	0.0235	0.0189	0.0131	0.0079
19800	population	0.0058	0.0103	0.0200	0.0202	0.0148	0.0103	0.0074

Size	Structure	OverallFoi	Foi1	Foi2	Foi3	Foi4	Foi5	Foi6
1650	survey	0.0053	0.0578	0.0453	0.0430	0.0355	0.0123	0.0120
3300	survey	0.0037	0.0423	0.0315	0.0298	0.0291	0.0098	0.0091
6600	survey	0.0027	0.0311	0.0241	0.0240	0.0212	0.0073	0.0057
9900	survey	0.0025	0.0244	0.0211	0.0199	0.0173	0.0055	0.0051
13200	survey	0.0021	0.0205	0.0160	0.0165	0.0139	0.0047	0.0048
19800	survey	0.0018	0.0167	0.0137	0.0144	0.0119	0.0040	0.0036
1650	uniform	0.0047	0.0692	0.0598	0.0575	0.0375	0.0141	0.0109
3300	uniform	0.0036	0.0484	0.0414	0.0457	0.0338	0.0108	0.0075
6600	uniform	0.0023	0.0339	0.0288	0.0300	0.0269	0.0082	0.0054
9900	uniform	0.0018	0.0291	0.0235	0.0244	0.0206	0.0063	0.0044
13200	uniform	0.0016	0.0251	0.0206	0.0216	0.0195	0.0051	0.0034
19800	uniform	0.0014	0.0197	0.0154	0.0173	0.0150	0.0044	0.0031
1650	population	0.0046	0.0806	0.0641	0.0583	0.0388	0.0138	0.0103
3300	population	0.0036	0.0527	0.0461	0.0474	0.0339	0.0104	0.0074
6600	population	0.0024	0.0377	0.0304	0.0297	0.0258	0.0082	0.0054
9900	population	0.0019	0.0309	0.0256	0.0259	0.0225	0.0065	0.0045
13200	population	0.0017	0.0266	0.0223	0.0224	0.0185	0.0056	0.0038
19800	population	0.0015	0.0214	0.0184	0.0189	0.0155	0.0046	0.0032

Table S12. Parvovirus B19 serological data – MSIRWb-ext AW model

Size	Structure	OverallPrev	Prev1	Prev2	Prev3	Prev4	Prev5	Prev6
1650	survey	0.0267	0.0078	0.0351	0.0440	0.0311	0.0430	0.0406
3300	survey	0.0194	0.0060	0.0273	0.0362	0.0207	0.0351	0.0297
6600	survey	0.0140	0.0036	0.0163	0.0219	0.0149	0.0243	0.0209
9900	survey	0.0122	0.0032	0.0147	0.0182	0.0124	0.0210	0.0192
13200	survey	0.0092	0.0030	0.0133	0.0163	0.0105	0.0203	0.0145
19800	survey	0.0083	0.0024	0.0107	0.0136	0.0091	0.0173	0.0118
1650	uniform	0.0203	0.0092	0.0415	0.0546	0.0345	0.0392	0.0252
3300	uniform	0.0155	0.0065	0.0301	0.0408	0.0260	0.0301	0.0180
6600	uniform	0.0109	0.0048	0.0221	0.0295	0.0177	0.0207	0.0135
9900	uniform	0.0081	0.0039	0.0180	0.0241	0.0133	0.0178	0.0102
13200	uniform	0.0070	0.0035	0.0159	0.0216	0.0125	0.0167	0.0086
19800	uniform	0.0056	0.0027	0.0123	0.0164	0.0096	0.0108	0.0077
1650	population	0.0197	0.0094	0.0427	0.0569	0.0358	0.0406	0.0235
3300	population	0.0151	0.0069	0.0316	0.0422	0.0260	0.0275	0.0167
6600	population	0.0101	0.0049	0.0222	0.0302	0.0178	0.0203	0.0126
9900	population	0.0086	0.0040	0.0182	0.0248	0.0147	0.0162	0.0103
13200	population	0.0070	0.0038	0.0175	0.0237	0.0128	0.0158	0.0086
19800	population	0.0057	0.0030	0.0137	0.0185	0.0102	0.0106	0.0078

Size	Structure	OverallFoi	Foi1	Foi2	Foi3	Foi4	Foi5	Foi6
1650	survey	0.0081	0.0169	0.0188	0.0160	0.0017	0.0048	0.0076
3300	survey	0.0064	0.0131	0.0146	0.0127	0.0014	0.0036	0.0060
6600	survey	0.0042	0.0079	0.0088	0.0076	0.0008	0.0022	0.0042
9900	survey	0.0039	0.0070	0.0078	0.0068	0.0007	0.0019	0.0040
13200	survey	0.0035	0.0064	0.0072	0.0062	0.0006	0.0018	0.0036
19800	survey	0.0025	0.0052	0.0058	0.0050	0.0005	0.0015	0.0025
1650	uniform	0.0095	0.0198	0.0220	0.0188	0.0020	0.0055	0.0079
3300	uniform	0.0068	0.0140	0.0155	0.0134	0.0014	0.0038	0.0059
6600	uniform	0.0051	0.0103	0.0114	0.0099	0.0011	0.0028	0.0045
9900	uniform	0.0042	0.0085	0.0095	0.0083	0.0009	0.0024	0.0039
13200	uniform	0.0038	0.0077	0.0086	0.0074	0.0008	0.0022	0.0037
19800	uniform	0.0028	0.0058	0.0065	0.0056	0.0006	0.0016	0.0024
1650	population	0.0099	0.0203	0.0225	0.0193	0.0020	0.0056	0.0085
3300	population	0.0072	0.0149	0.0165	0.0142	0.0015	0.0041	0.0061
6600	population	0.0053	0.0107	0.0118	0.0102	0.0011	0.0030	0.0047
9900	population	0.0042	0.0086	0.0096	0.0083	0.0009	0.0024	0.0035
13200	population	0.0041	0.0083	0.0093	0.0081	0.0009	0.0023	0.0037
19800	population	0.0031	0.0064	0.0072	0.0062	0.0007	0.0018	0.0026

Size	Structure	R_0	R_{eff}	ASI	φ
1650	survey	0.4543	0.0088	1.2815	1.0859
3300	survey	0.3533	0.0069	1.0170	0.4921
6600	survey	0.2126	0.0042	0.6099	0.3999
9900	survey	0.1882	0.0037	0.5419	0.3306
13200	survey	0.1732	0.0034	0.4963	0.3162
19800	survey	0.1406	0.0027	0.3992	0.1803
1650	uniform	0.5320	0.0103	1.5003	0.4664
3300	uniform	0.3756	0.0073	1.0588	0.3797
6600	uniform	0.2757	0.0054	0.7776	0.2191
9900	uniform	0.2298	0.0045	0.6626	0.2534
13200	uniform	0.2067	0.0040	0.5953	0.1764
19800	uniform	0.1573	0.0031	0.4478	0.0994
1650	population	0.5448	0.0106	1.5179	0.5419
3300	population	0.3988	0.0078	1.1245	0.3904
6600	population	0.2861	0.0056	0.8054	0.1948
9900	population	0.2315	0.0045	0.6633	0.1386
13200	population	0.2245	0.0044	0.6458	0.1613
19800	population	0.1735	0.0034	0.4946	0.0994

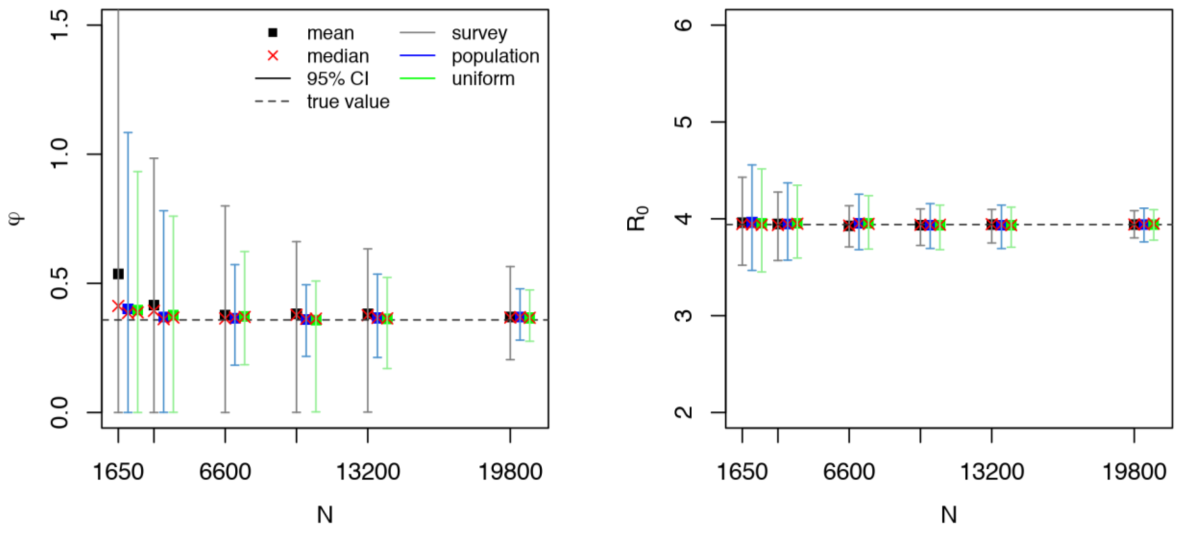

Figure S4. Parvovirus B19 serological data: mean, median, and 95% confidence interval for the relative boosting factor φ (left) and basic reproduction number R_0 (right) over 500 simulations as a function of the total number of sampled individuals (N) for the MSIR model allowing for age-specific waning of disease-acquired antibodies and boosting of low immunity (MSIRWb-ext AW) model.
"True" value is the value estimated using the model on the observed serological data (with integer age values). The y-axes have different ranges of values for better legibility.

Optimal distributions

In Tables S13-20, the optimal distributions of samples for the overall and age-specific seroprevalence and force of infection by pathogen, model, and sample size are given. The age groups (Group 1-6) are [1,2), [2,6), [6,12), [12,19), [19,31), and [31,65] years.

Table S13. Measles serological data – Logistic model with piecewise constant prevalence

Size	Group1 %	Group2 %	Group3 %	Group4 %	Group5 %	Group6 %	Precision
**Overall prevalence**							
1650	10	10	10	20	20	30	0.0104
3300	10	20	10	10	20	30	0.0081
6600	10	10	10	20	20	30	0.0053
9900	10	10	10	10	30	30	0.0042
13200	10	10	10	20	20	30	0.0036
19800	10	10	10	20	20	30	0.0031
**Prevalence by age group**							
1650	20	20	10	20	20	10	0.1950
3300	30	10	20	10	20	10	0.1332
6600	20	20	10	20	20	10	0.0953
9900	20	20	10	20	20	10	0.0759
13200	30	10	20	10	20	10	0.0675
19800	20	10	20	10	20	20	0.0567

Table S14. Mumps serological data – Logistic model with piecewise constant prevalence

Size	Group1 %	Group2 %	Group3 %	Group4 %	Group5 %	Group6 %	Precision
**Overall prevalence**							
1650	10	10	10	10	10	50	0.0191
3300	10	20	10	10	10	40	0.0143
6600	10	10	10	10	10	50	0.0097
9900	10	10	10	10	20	40	0.0080
13200	10	10	10	10	10	50	0.0070
19800	10	10	10	20	10	40	0.0062
**Prevalence by age group**							
1650	20	20	10	10	20	20	0.2642
3300	20	10	30	10	20	10	0.1877
6600	20	20	10	10	20	20	0.1289
9900	20	10	20	10	20	20	0.1041
13200	20	10	20	10	20	20	0.0916
19800	20	20	10	10	20	20	0.0752

Table S15. Rubella serological data – Logistic model with piecewise constant prevalence

Size	Group1 %	Group2 %	Group3 %	Group4 %	Group5 %	Group6 %	Precision
**Overall prevalence**							
1650	10	10	10	20	10	40	0.0106
3300	10	10	10	20	10	40	0.0077
6600	10	10	10	10	20	40	0.0054
9900	10	10	10	10	30	30	0.0044
13200	10	10	10	10	20	40	0.0037
19800	10	10	10	20	10	40	0.0031
**Prevalence by age group**							
1650	20	20	20	10	10	20	0.1793
3300	30	10	20	10	20	10	0.1269
6600	20	20	20	10	10	20	0.0873
9900	30	20	10	10	10	20	0.0735
13200	30	10	30	10	10	10	0.0635
19800	30	10	20	10	20	10	0.0510

Table S16. VZV serological data – Exponentially damped model

Size	Group1 %	Group2 %	Group3 %	Group4 %	Group5 %	Group6 %	Precision
**Overall prevalence**							
1650	30	10	10	30	10	10	0.0048
3300	10	30	10	10	30	10	0.0050
6600	10	10	10	10	40	20	0.0020
9900	10	10	10	10	40	20	0.0021
13200	50	10	10	10	10	10	0.0020
19800	10	10	20	10	30	20	0.0019
**Prevalence by age group**							
1650	20	20	20	20	10	10	0.1081
3300	20	40	10	10	10	10	0.0859
6600	10	20	10	40	10	10	0.0585
9900	10	20	10	10	30	20	0.0520
13200	10	30	20	10	10	20	0.0420
19800	20	20	30	10	10	10	0.0342
**Overall force of infection**							
1650	20	20	20	10	20	10	0.0264
3300	10	30	10	10	30	10	0.0255
6600	10	10	10	10	40	20	0.0094
9900	10	10	10	10	40	20	0.0101
13200	50	10	10	10	10	10	0.0096
19800	10	10	10	20	30	20	0.0096
**Force of infection by age group**							
1650	10	20	10	10	30	20	0.2593
3300	10	20	20	10	20	20	0.1842
6600	10	20	20	20	10	20	0.1208
9900	10	20	10	10	10	40	0.0991
13200	10	30	20	10	10	20	0.0883
19800	20	20	20	10	10	20	0.0696

Table S17. VZV serological data – MSIR piecewise constant force of infection

Size	Group1 %	Group2 %	Group3 %	Group4 %	Group5 %	Group6 %	Precision
**Overall prevalence**							
1650	10	20	10	10	30	20	0.0069
3300	10	20	20	10	30	10	0.0048
6600	10	20	10	10	30	20	0.0032
9900	10	10	20	10	30	20	0.0026
13200	10	10	20	10	30	20	0.0024
19800	10	10	20	10	30	20	0.0020
**Prevalence by age group**							
1650	10	40	10	20	10	10	0.1229
3300	20	30	20	10	10	10	0.0914
6600	10	40	10	20	10	10	0.0630
9900	10	50	10	10	10	10	0.0538
13200	10	30	20	10	20	10	0.0464
19800	10	40	20	10	10	10	0.0374
**Overall force of infection**							
1650	10	10	40	10	10	20	0.0356
3300	10	10	30	10	30	10	0.0266
6600	10	10	30	10	20	20	0.0187
9900	10	10	20	10	20	30	0.0144
13200	10	10	20	10	30	20	0.0132
19800	10	10	20	10	30	20	0.0106
**Force of infection by age group**							
1650	10	40	10	10	20	10	0.5954
3300	10	30	10	30	10	10	0.5147
6600	10	20	10	10	10	40	0.3494
9900	10	10	20	10	10	40	0.2953
13200	10	10	20	20	10	30	0.2529
19800	10	10	20	10	20	30	0.1976

Table S18. Parvovirus B19 serological data – Exponentially damped model

Size	Group1 %	Group2 %	Group3 %	Group4 %	Group5 %	Group6 %	Precision
**Overall prevalence**							
1650	10	10	20	10	20	30	0.0214
3300	10	10	10	20	10	40	0.0146
6600	10	10	10	20	10	40	0.0107
9900	10	10	10	10	20	40	0.0088
13200	20	10	10	10	20	30	0.0074
19800	10	10	10	10	20	40	0.0063
**Prevalence by age group**							
1650	10	20	10	20	20	20	0.1725
3300	10	20	10	20	10	30	0.1258
6600	10	10	30	10	20	20	0.0888
9900	10	30	10	20	10	20	0.0748
13200	10	20	20	10	10	30	0.0634
19800	10	20	20	10	20	20	0.0536
**Overall force of infection**							
1650	10	10	10	10	10	50	0.0043
3300	10	10	10	10	10	50	0.0030
6600	10	10	10	10	10	50	0.0021
9900	10	10	10	10	10	50	0.0018
13200	10	10	10	10	10	50	0.0015
19800	10	10	10	10	10	50	0.0014
**Force of infection by age group**							
1650	10	30	10	20	10	20	0.0630
3300	10	30	10	20	10	20	0.0458
6600	10	40	10	10	10	20	0.0329
9900	10	30	10	10	20	20	0.0264
13200	10	30	10	10	20	20	0.0232
19800	10	20	20	10	20	20	0.0195

Table S19. Parvovirus B19 serological data – MSIR piecewise constant force of infection

Size	Group1 %	Group2 %	Group3 %	Group4 %	Group5 %	Group6 %	Precision
**Overall prevalence**							
1650	10	10	20	10	20	30	0.0212
3300	10	10	10	10	20	40	0.0147
6600	10	10	10	10	30	30	0.0106
9900	10	10	10	10	20	40	0.0087
13200	10	10	10	10	20	40	0.0072
19800	10	10	10	10	10	50	0.0064
**Prevalence by age group**							
1650	10	20	20	10	20	20	0.2059
3300	10	10	20	10	20	30	0.1527
6600	10	20	20	20	10	20	0.1086
9900	10	30	10	20	10	20	0.0869
13200	10	20	20	10	20	20	0.0754
19800	10	20	20	20	10	20	0.0628
**Overall force of infection**							
1650	10	10	10	10	10	50	0.0047
3300	10	10	10	10	10	50	0.0033
6600	10	10	10	10	10	50	0.0022
9900	10	10	10	10	10	50	0.0020
13200	10	10	10	10	10	50	0.0017
19800	10	10	10	10	10	50	0.0015
**Force of infection by age group**							
1650	10	40	10	10	10	20	0.1692
3300	20	20	10	20	10	20	0.1299
6600	10	20	20	10	20	20	0.0936
9900	10	20	30	10	20	10	0.0762
13200	10	20	40	10	10	10	0.0669
19800	10	20	30	10	10	20	0.0536

Table S20. Parvovirus B19 serological data – MSIRWb-ext AW model

Size	Group1 %	Group2 %	Group3 %	Group4 %	Group5 %	Group6 %	Precision
**Overall prevalence**							
1650	10	10	10	20	20	30	0.0237
3300	10	10	10	10	10	50	0.0156
6600	10	10	10	10	20	40	0.0115
9900	10	10	10	10	20	40	0.0090
13200	10	10	10	10	20	40	0.0075
19800	10	10	10	10	10	50	0.0067
**Prevalence by age group**							
1650	10	10	20	20	20	20	0.1921
3300	10	10	20	10	10	40	0.1435
6600	10	10	10	20	20	30	0.1029
9900	10	10	20	10	10	40	0.0817
13200	10	10	20	10	10	40	0.0740
19800	10	20	20	10	10	30	0.0615
**Overall force of infection**							
1650	10	20	20	20	10	20	0.0076
3300	10	10	30	10	20	20	0.0058
6600	10	20	20	10	10	30	0.0042
9900	10	10	20	10	10	40	0.0035
13200	10	10	20	10	10	40	0.0030
19800	10	10	20	30	10	20	0.0023
**Force of infection by age group**							
1650	10	10	30	20	10	20	0.0596
3300	10	10	30	10	20	20	0.0444
6600	10	20	20	10	10	30	0.0339
9900	10	10	20	20	10	30	0.0265
13200	10	10	20	10	10	40	0.0245
19800	10	10	20	30	10	20	0.0184

References

Goeyvaerts N, Hens N, Aerts M, Beutels P. Model structure analysis to estimate basic immunological processes and maternal risk for parvovirus B19. Biostatistics. 2011; 12: 283-302.

Goeyvaerts N, Hens N, Ogunjimi B, Aerts M, Shkedy Z, Van Damme P, Beutels P. Estimating infectious disease parameters from data on social contacts and serological status. Journal of the Royal Statistical Society Series C-Applied Statistics. 2010; 59: 255-77.

Santermans E, Goeyvaerts N, Melegaro A, Edmunds WJ, Faes C, Aerts M, Beutels P, Hens N. The social contact hypothesis under the assumption of endemic equilibrium: Elucidating the transmission potential of VZV in Europe. Epidemics. 2015; 11: 14-23.
